# Biomimetically engineered Amphotericin B nano-aggregates circumvent toxicity constraints and treat systemic fungal infection in experimental animals

**DOI:** 10.1038/s41598-017-11847-0

**Published:** 2017-09-19

**Authors:** Qamar Zia, Owais Mohammad, Mohd Ahmar Rauf, Wasi Khan, Swaleha Zubair

**Affiliations:** 1 Interdisciplinary Biotechnology Unit, Aligarh Muslim University, Aligarh, India; 2 Department of Applied Physics, Aligarh Muslim University, Aligarh, India; 3 Women’s College, Aligarh Muslim University, Aligarh, India

## Abstract

Biomimetic synthesis of nanoparticles offers a convenient and bio friendly approach to fabricate complex structures with sub-nanometer precision from simple precursor components. In the present study, we have synthesized nanoparticles of Amphotericin B (AmB), a potent antifungal agent, using *Aloe vera* leaf extract. The synthesis of AmB nano-assemblies (AmB-NAs) was established employing spectro-photometric and electron microscopic studies, while their crystalline nature was established by X-ray diffraction. AmB-nano-formulation showed much higher stability in both phosphate buffer saline and serum and exhibit sustained release of parent drug over an extended time period. The as-synthesized AmB-NA possessed significantly less haemolysis as well as nephrotoxicity in the host at par with Ambisome^®^, a liposomized AmB formulation. Interestingly, the AmB-NAs were more effective in killing various fungal pathogens including *Candida* spp. and evoked less drug related toxic manifestations in the host as compared to free form of the drug. The data of the present study suggest that biomimetically synthesized AmB-NA circumvent toxicity issues and offer a promising approach to eliminate systemic fungal infections in Balb/C mice.

## Introduction

Among various available antifungals, Amphotericin B (AmB) is considered as the drug of choice in therapy of life-threatening systemic fungal infections^1^. Unfortunately, the clinical usefulness of AmB and its conventional formulation (Fungizone^®^) is restricted due to infusion-associated febrile reactions^2^ as well as dose-dependent nephrotoxicity in the host^3^. To circumvent toxicity related constraints associated with AmB^4^, several lipid-based delivery systems have been introduced^5^. The liposome based AmB formulation (AmBisome^®^) permits use of relatively large doses of the drug with much lower side effects and toxicity^5^. However, concerns like high production cost, shelf-life related issues, necessity of repeated intra-venous injections for successful treatment^6–9^ *etc*. impede the widespread use of this gold standard drug. Some earlier attempts to reduce AmB toxicity resulted in substantial decline in its biological activity^10–12^. Such developments compel us to look for development of more efficaious alternate AmB formulation.

Both toxic and chemotherapeutic effects of AmB have a strong correlation to its aggregation state^13,14^. AmB has very poor solubility in both polar (<1 µg/ml) as well as in organic solvents such as cyclohexane (20 µg/mL); and tends to self-associate with a critical aggregation concentration (CAC) of 1 µg/ml^15^. The unique physico-chemical property of AmB also helps in its intercalation into plasma membranes of host cells facilitating pore formation^16^. AmB exist in the form of large aggregates both in water as well as the lipid membrane. A new poly-aggregated form of AmB developed by mild heating of Fungizone^15^, has been shown to exhibit better efficacy in treatment of experimental leishmaniasis^17^. Compared to water soluble oligomeric aggregates present in the Fungizone, the AmB super-aggregates exhibited considerably reduced toxicity to mammalian cells *in vitro* and to mice *in vivo* ^18–20^. It is, therefore plausible, that the cytotoxicity of AmB could simply be circumvented by controlling the degree of aggregation.

Biomimetic approach to induce synthesis of pre-defined nano-structured entities from simple monomeric precursors is a simple, eco-friendly and economically feasible process^21^. The approach has been widely exploited for synthesis of metal based nanoparticles and offers an attractive strategy to fabricate nano-sized dosage forms^22–25^, especially those of therapeutically important drugs_._ Nano-sized drug assemblies devoid of any pharmaceutical carrier (excipients) are more discrete, since they avoid excipient related limitations^26^. In spite of greater implications, there is limited advancement in the biologically inspired synthesis of organic molecules based nanoparticles.

Several plants, bacteria and fungi have been reported to possess tremendous potential in synthesis of metal based nanoparticles^22,27,28^. The use of plants based materials in the synthesis of nanoparticles is economical, eco-friendly and eliminates the elaborate process of maintaining microbial cultures^29^. Potential of plethora of plants such as *Cinnmonum camphora* ^29^, *Aloe vera* ^30^, *Chrysopogon zizanioides* ^31^, *Azadirachta indica* ^32^, *Rumex hymenosepalus* ^33^, *Citrus sinensis* ^34^, *Ocimum sanctum* ^35^ in fabricating nano-structured entities have been well documented. However, the ability of plant extract to fabricate nano-assembly of antifungal drugs has not been explored yet. Taking a lead from our earlier work on the biomimetic synthesis of nucleobase nano-assemblage^36^, we envisaged *Aloe vera* leaf extract mediated biofabrication of AmB nanoparticles or aggregates employing the same strategy. The as-synthesized super-aggregated AmB nano-assembly (AmB-NA) was characterised by TEM, XRD, and several related spectro-photometric techniques. The super aggregated AmB-NAs displayed markedly reduced toxicity without compromising its antifungal activity. Furthermore, as-synthesized AmB-NA successfully treated systemic *C*. *albicans* infection in Balb/C mice.

## Results

### Spectroscopic analysis of AmB - NAs

**Figure 1:**
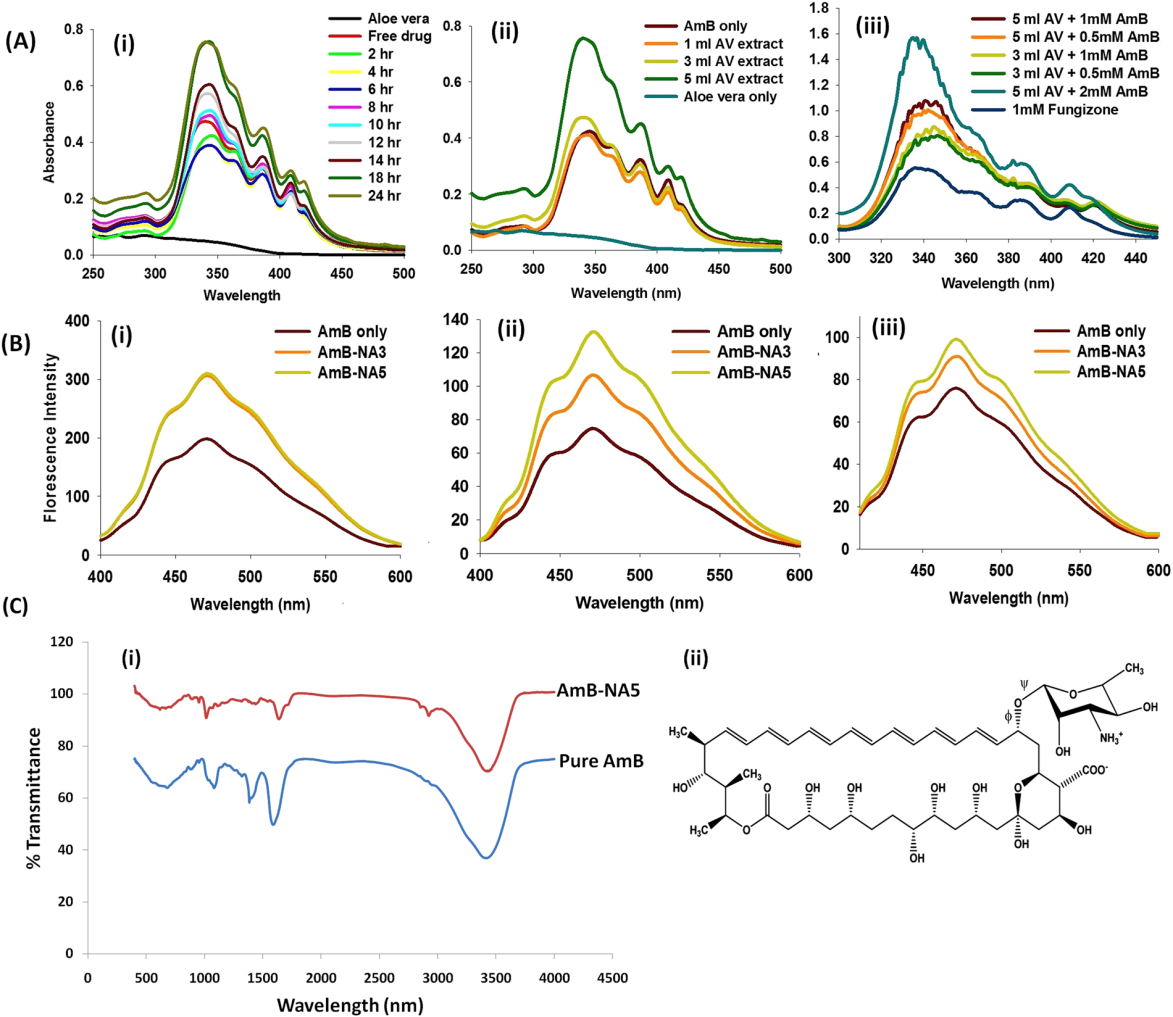
(**A**) Aloe vera leaf extract induces biomimetic synthesis of AmB-NAs: Effect of various parameters on biomimetic synthesis of AmB-NA (i) Time kinetics of AmB-NA synthesis (ii) UV-VIS spectra of AmB-NA to monitor interaction of 1 mM AmB solution with varying volume of *Aloe vera* leaf extract (iii) Effect of increasing concentration of AmB on biomimetic synthesis of AmB-NA as revealed by UV-VIS spectroscopy. (**B**) Biomimetically synthesized AmB-NA exhibits superaggregated assembly pattern: Fluorescence emission (Em) spectra of AmB-NA as well as Fungizone at (i) 408 nm (ii) 250 nm (iii) 325 nm. (**C**) The characteristic functional groups of parent AmB compound remained intact in as synthesized AmB-NA: (i) FTIR absorption spectra of AmB formulations (ii) Structure of AmB.

The spectroscopic analysis revealed that as-synthesized AmB-NAs retained UV-absorption characteristics of parent AmB compound. The UV-VIS spectra of as-aynthesized nano-particles resembled closely to that of super-aggregates of AmB (i.e a hypsochromic shift in the λ_max_ with highest peak ca 340 nm). This characteristic spectrum was monitored throughout the study. For the sake of simplicity, the absorption spectrum of the free AmB (pure drug) scanned just after its mixing with *Aloe vera* leaf extract was annotated as zero time point observation. The interaction of AmB with *Aloe vera* leaf contents resulted in quenching in the absorbance intensity of various characteristic absorption peaks of the AmB upto the time period of 6 hours (Fig. 1A(i)). A significant increase in intensity was observed after 6 hours; which was greater than the intensity of free AmB at zero time point. The process of super-assembly (nanoparticles) formation was slow and gets completed in 18 hours as longer incubation (beyond 24 hours) did not result in any further increase in absorbance. Nanoassemblies (NAs) thus formed, were pelleted by centrifugation and washed several times to remove traces of *Aloe vera* extract.

The effect of increasing concentration of *Aloe vera* leaf extract on super-aggregation of nanoparticle synthesis was also studied. At a fixed time point (18 h), insignificant decrease was found in the absorbance upon addition of 1 ml *Aloe vera* leaf extract to 5 ml of 1 mM AmB solution (final reaction mixture volume 10 ml) (Fig. 1A(ii)). However, mixing of AmB with relatively larger ratio of *Aloe vera* extract (3 ml leaf extract mixed with 5 ml of 1 mM AmB solution; final volume 10 ml) brings about an increase in absorbance. The resulting nanoparticles were annotated as AmB-NA3. Further increment in *Aloe vera* leaf extract (5 ml) resulted in an abrupt increase in peak intensity, significantly higher than the original peak of free AmB. The nanoparticles formed upon employing 5 ml of *Aloe vera* extract (keeping final reaction volume to 10 ml), were referred to as AmB-NA5. A hypsochromic shift in the absorption spectrum of as-synthesized AmB-NAs (super aggregates) was observed upon incubation with increasing volumes of *Aloe vera* leaf extract. This suggests that increased concentration of *Aloe vera* leaf extract is more actively involved in formation of drug nano-assembly. Effect of variation in the AmB concentration (0.5–2 mM AmB equivalent) on nano-assembly formation was also studied. Formation of nano-aggregates was found to be directly proportional to initial AmB content (Fig. 1A(iii)). Highest aggregate formation was observed with 2 mM followed by 1 mM AmB solution. The solution with 0.5 mM AmB concentration displayed lowest nano-drug formation as evidenced by intensities of various peaks in the corresponding spectrum.

The UV absorption spectra were recorded both for aqueous suspension of AmB nano-particles as well as its free form (Figure S1). AmB absorption spectra in DMSO was characteristic of monomers with the minor peak at 355 nm and major peak at 415 nm (Figure S1A); with 0–0, 0–1 and 0–2 vibrational maxima corresponding to 408, 383 and 371 nm, respectively. The principal absorbance peak of AmB-NA in water was found to be around 340 nm, while minimal peak was observed at 410 nm (Figure S1B). Among the two, AmB-NA5 exhibited higher absorptivity as compared to AmB-NA3.

### Fluorescence spectral analysis

At 408 nm, where monomeric form of AmB absorbs principally, the fluorescence intensity of Fungizone was much higher when compared to AmB-NA3 or AmB-NA5 (Fig. 1B(i)). The fluorescence intensity corresponding to dimer excitation (350 nm) increased for nano-assembled form of AmB. A strong fluorescence peak at 472 nm was observed indicating the presence of the dimeric form (Fig. 1B(ii)). The vibrational substructure, viz. 0–0, 0–1 and 0–2, can be assigned to the observed maxima at 442, 471 and 502 nm, respectively. Florescence emission spectrum of AmB-NA solutions at 325 nm (characteristic of the super-aggregated species)^37^ exhibited a peak at 448 nm and a shoulder at 463 nm (Fig. 1B(iii)), with much higher intensity for AmB-NA5 than for AmB-NA3.

### FT-IR Spectra analysis

The IR spectra of AmB-NAs (super aggregates) showed characteristic peaks of the parent drug (Fig. 1C(i)). The spectral region between 1500 and 1800 cm^−1^ represents the stretching vibrations of the C=O, —COO^−^, —NH_3_^+^ and C = C functional groups (Table S1). There is increase of the band width in the range 3300–3500 cm^−1^, characteristic for hydrogen-bonded AmB molecules (—OH••••HO—). The formation of molecular pores is related to the influence of the hydroxyl groups, where corresponding bands appear in the region of deformational vibrations of the C—O bond. The bands in the range of 1330–1040 cm^−1^ are associated with stretching vibrations (deformation of C—O and C—O—C bonds) as well as C—H (deformation of out-of-plane vibrations) in the AmB chromophores characteristic of all-trans polyenes^38^, which further indicates their participation in the nano-assembly formation. A sharp intense band centered at 1604, and 1657 cm^−1^ is clearly visible (Gagos’ and Arczewska 2010, 2011). This is a direct indication of the asymmetric stretch of the –COO- group in AmB^39^. The structure of Amphotericin B has been provided in Fig. 1C(ii) for better understanding of the FTIR peaks.

### Physico-chemical and surface properties of AmB-NAs

**Figure 2:**
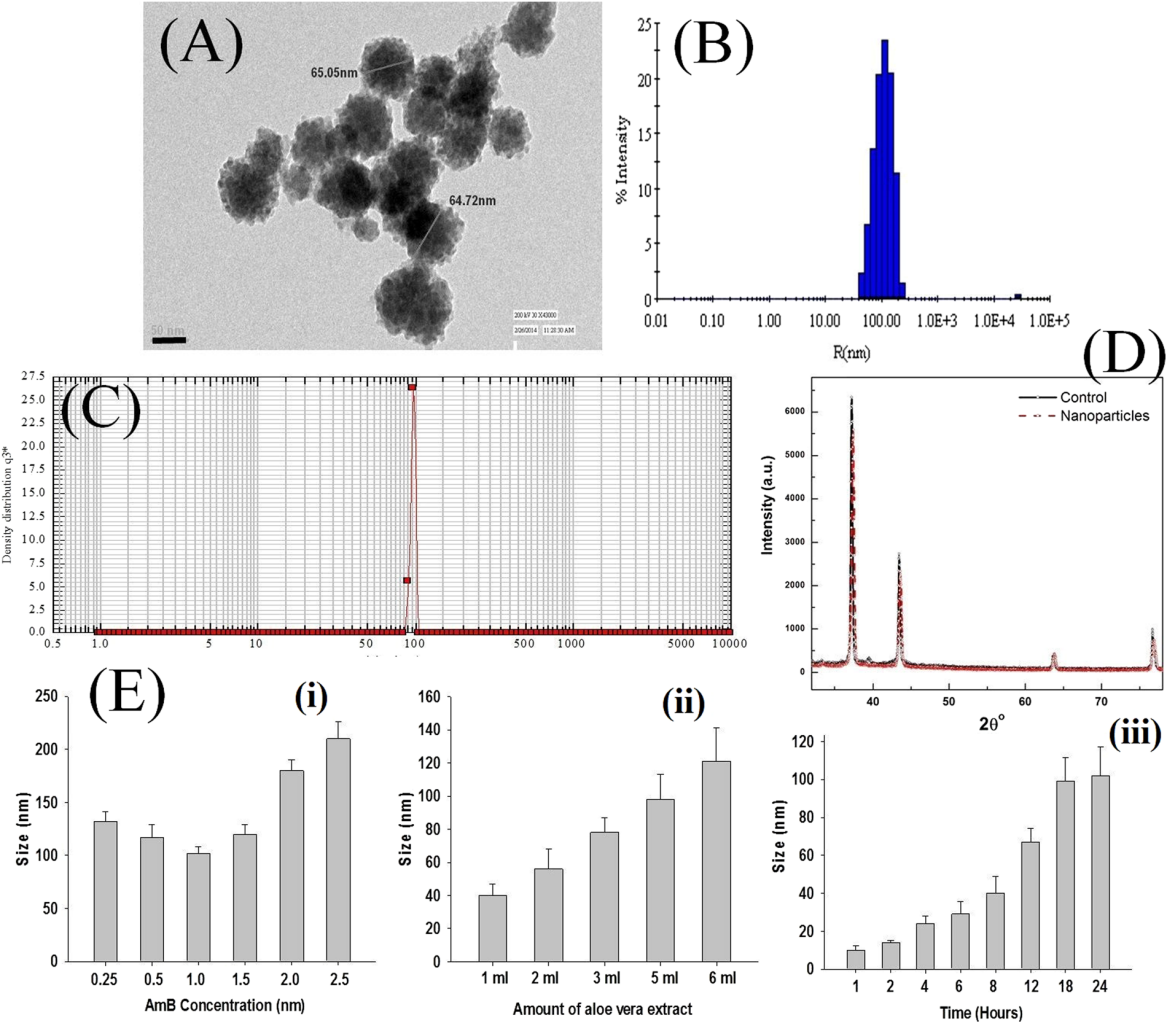
Size distribution and morphology of AmB-NA: (**A**) Representative TEM image of optimized AmB-NA synthesized by employing 5 ml of *Aloe Vera* leaf extract mixed with 5 ml of 1 mM AmB solution. (**B**) The average diameter of AmB-NA determined by DLS measurements was 95 ± 12 nm and 104 ± 9 nm for AmB-NA3 and AMB-NA5. (**C**) Particle size distribution of AmB-NA assessed by photon correlation spectroscopy. (**D**) XRD pattern of AmB-NA. (**E**) Effect of change in concentration of (i) AmB (ii) *Aloe vera* leaf extract and (iii) reaction time on the size of the AmB-NA.

The representative TEM image shows particles to be nearly spherical with uneven surface morphology and narrow size distribution, having sizes 62 ± 6 and 65 ± 9 nm for AmB-NA3 and AmB-NA5 respectively (Fig. 2A). The particle size distribution in aqueous medium measured by dynamic light-scattering was 95 ± 12 nm and 104 ± 9 nm (Fig. 2B) for AmB-NA3 and AmB-NA5 respectively. The average zeta diameter of AmB-NA3 was found to be 100 ± 8 nm, while it was 102 ± 5 nm for AmB-NA5 (Fig. 2C). The zeta potential value for AmB-NA3 and AmB-NA5 was found to be −29.6 ± 2.1 and −34.2 ± 5.7 mV respectively (Table 1). Figure 2D shows X-ray diffraction (XRD) patterns of pure AmB and AmB-NAs. All the observed diffraction peaks can be indexed using standard data, which exhibits clear crystalline nature of the nanoparticles. Moreover, no impurity peaks were detected in the patterns, which revealed the high phase purity of the samples. The 2θ value of the most intense peak had been observed at ~37°.

**Table 1:** Size and zeta potential of various AmB nano-formulations.

Formulation	Average Zeta Diameter (nm)	Zeta potential (mV)	D_h_^a^ (nm)	PDI^b^
AmB-NA3	100 ± 8	−29.6 ± 2.1	95 ± 12	0.15 ± 0.04
AmB-NA5	102 ± 5	−34.2 ± 5.7	104 ± 9	0.22 ± 0.03

^a^Volume-averaged hydrodynamic diameter (D_h_) ± standard deviation.

^b^Polydispersity Index ± standard deviation.

The size-dimension of AmB-NAs was evaluated after varying one parameter at a time; while keeping other parameters constant (Fig. 2E). When the effect of varying AmB concentration on the size of AmB-NA was studied, two different patterns of nanoparticle size evolution were obtained. At 1 mM AmB concentration, a general decrease in particle size was observed with increase in AmB concentration, while the NAs synthesized using AmB concentrations ≥1 mM showed an overall increase in diameter with increase in AmB concentration (Fig. 2E(i)). We also observed that increment in the content of *Aloe vera* leaf extract led to the increase in the size of the as synthesized NAs (Fig. 2E(ii)). Time kinetics studies revealed an increment in the size of the nanoparticles with time; maximum size was obtained on the completion of the reaction i.e. after 24 hours (Fig. 2E(iii)). There was insignificant difference in the size of nanoparticles at 18 and 24 hr.

### Release kinetics of monomeric AmB from AmB-NAs

**Figure 3:**
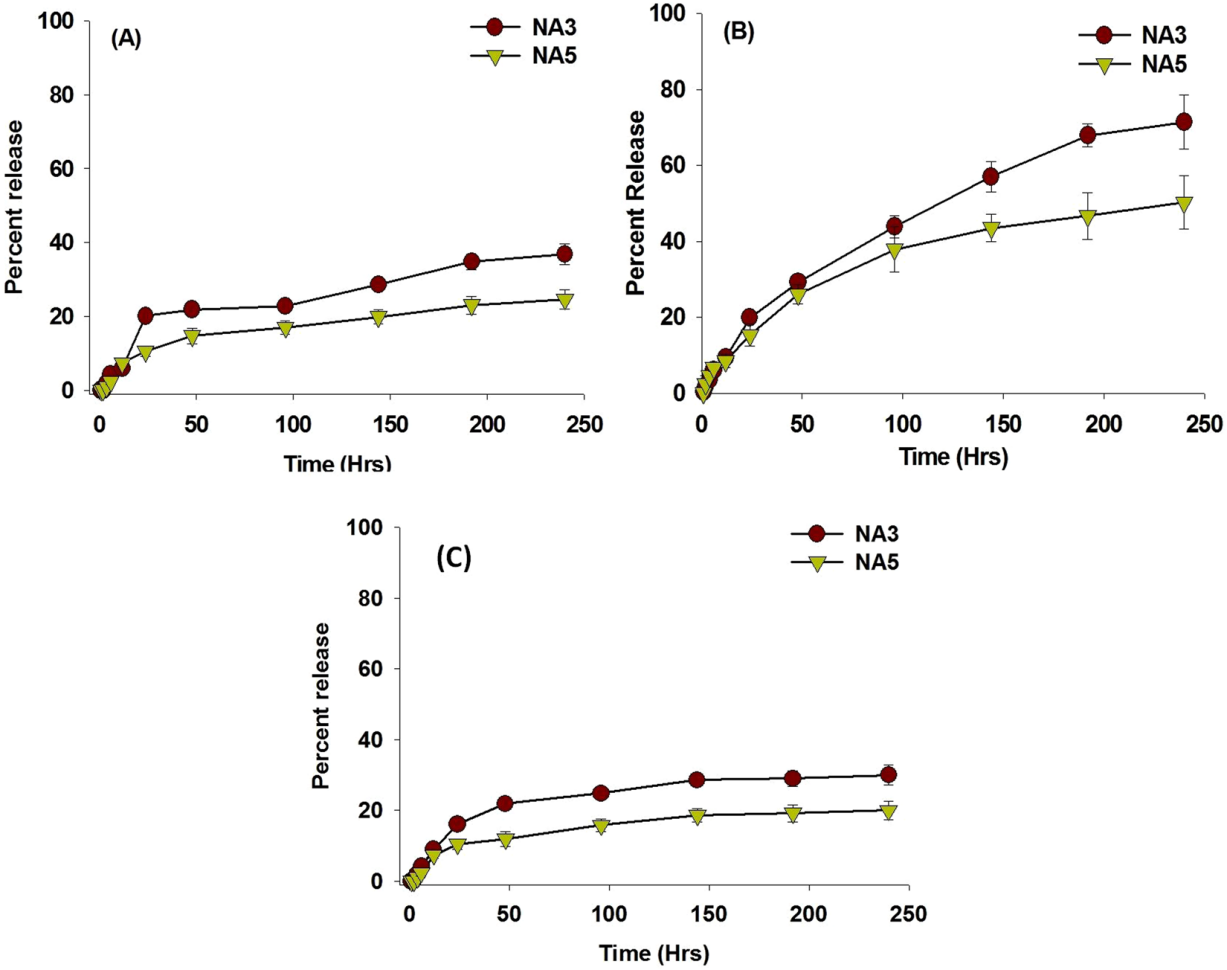
AmB-NA releases monomeric AmB in sustained manner over extended time period: Various AmB-NA formulations were incubated in (**A**) sterile 20 mM phosphate buffer saline (pH 7.4) (**B**) Serum. (**C**) Histidine Buffer. The amount of AmB released at various time points was analysed spectrophotometrically at 405 nm. Each time point represents an average of three runs ± SD.

The nano-assemblies exhibited a biphasic release pattern (Fig. 3), consisting of a small initial burst followed by a sustained and continuous release of the parent drug over an extended time period. Approximately 16% and 10% of the total drug was released in PBS within the first 24 hr from the AmB-NA3 and AmB-NA5 formulations respectively. Subsequently, both the NAs exhibited a controlled release pattern over a period of ∼240 hr (Fig. 3A). AmB-NA5 exhibited greater stability in serum when compared to AmB-NA3; A total of 75.21 ± 2.09% and 56.41 ± 1.02% of AmB content was released from AmB-NA5 and AmB-NA3 respectively after 240 hr (Fig. 3B). Moreover, the nanoparticles were found to be stable in histidine buffer resulting in release of less than 30% of the total drug over 240 hr time period (Fig. 3C).

### *In vitro* toxicity tests

**Figure 4:**
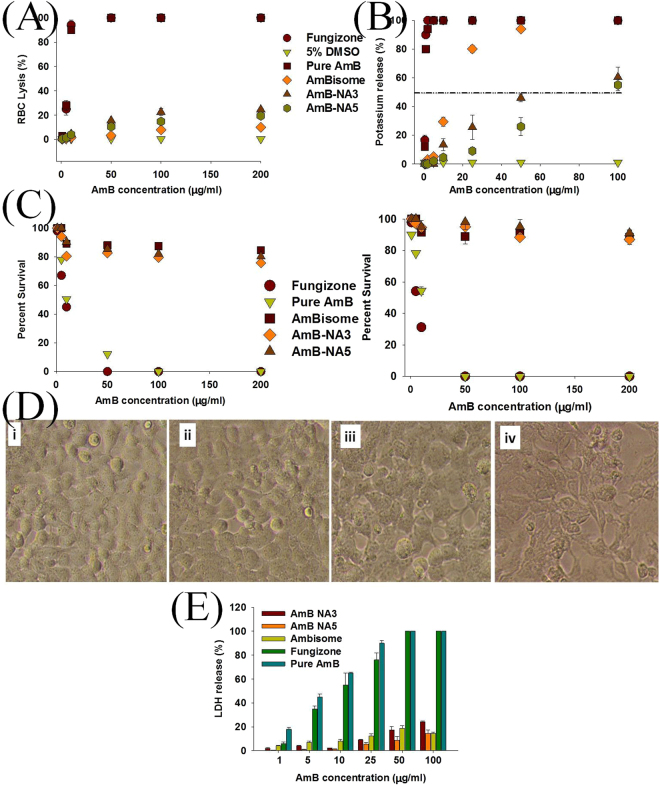
*In vitro* toxicity tests: (**A**) Hemolysis caused by AmB-NA upon their interaction with human RBCs. The extent of damage incurred to blood erythrocytes by AmB-NA was measured as percentage lysis of total erythrocytes (**B**) Intracellular K^+^ leakage incurred by human RBCs upon exposure to various AmB formulations. (**C**) Dose-response effects of AmB nano-formulation on cytotoxicity against (a) HEK-293cells (b) J774A.1 cells. The cells were exposed to two forms of AmB-NA for 24 hr. MTT values were normalized to the control untreated cells. Data are reported as means ± standard deviation of quadruplet. Fungizone, AmBisome and pure AmB were taken as controls. (**D**) Representative photomicrograph of J774A.1 cell line treated with (i) 25 µg/mL, (ii) 50 µg/mL, (iii) 100 µg/mL and (iv) 200 µg/mL concentration of AmB-NA for 24 hr. (**E**) LDH release induced by various AmB formulations, as a function of membrane damage in macrophage J774 A.1. The LDH release was detected by measuring the absorbance of colored complex at 500 nm. Triton X-100 (0.1%) was used as a positive control. Fungizone, AmBisome and pure AmB powder used in preparation of the complex were taken as controls. Data represented are pooled from three different experiments. Each data point is an average ± SD. *Aloe vera* leaf extract showed no change in any of the parameters tested (data not shown).

The lysis of erythrocytes upon interaction with AmB-NA formulations is shown in Fig. 4A. The hemoglobin leakage is depicted as percentage of total lysis (100%) induced by exposure of cells to Triton X-100. Fungizone was the most toxic formulation for the RBCs at all concentrations tested. In case of either AmB-NA3 or AmB-NA5, no significant hemolysis was observed at low concentration (1 µg/ml).. At a concentration of 200 μg/ml AmB equivalent, 24.6 ± 1.5% and 19.4 ± 2.2% hemolysis was observed in case of AmB-NA3 and AmB-NA5 formulations respectively. AmBisome, used as a control, displayed minimal hemolysis (maximum leakage of hemoglobin was 10.0 ± 0.6% at a concentration of 200 μg/ml AmB equivalents). The two as-synthesized nanoformulations exhibited Hb_50_ (50% hemolysis) above the highest concentration tested (200 μg/ml). The positive control, Fungizone, induced a sharp increase in toxicity as the concentration was increased from 5 µg/ml to 10 µg/ml (25.0 ± 5.0% and 94.0 ± 4.3% respectively); with complete hemolysis at 50 µg/ml AmB concentration and above (Fig. 4A). Free form of the drug induced same extent of toxicity as that of Fungizone.

The efflux of K^+^, a parameter to study leakage across plasma membrane, suggested significant differences between Fungizone and AmB-NA toxicity (p < 0.001). At a concentration of 50 µg/ml, AmB-NA5 induced significantly lower K^+^ leakage (26.0 ± 6.3%), while Fungizone induced 100% K^+^ leakage. The K_50_ (50% K^+^ leakage) measurement for AmBisome was about 15.8 µg/mL, which was 20-fold greater than the K_50_ values for Fungizone (K_50_ = 0.77 µg/mL) (Fig. 4B). As determined from the sigmoidal fit curve of the experimental data, the AmB-NA concentration required to trigger 50% K^+^ leakage was about 61 and 90 µg/mL for AmB-NA3 and AmB-NA5 formulations, respectively.

Next, the cytotoxic effect of AmB-NA formulation was assessed against mammalian cell lines viz. HEK-293 (Human embryonic kidney cells 293) and J774A.1 macrophage-like cells (Fig. 4C and D). Both AmBisome and AmB-NA based formulations were least toxic with an IC_50_ (half maximal inhibitory concentration) value above 100 mg/L after 24 h exposure (Table 2) while Fungizone exhibited dose dependent toxicity (Fig. 4C). AmB concentration as high as 200 μg/mL resulted in survival of 75.7 ± 1.5% and 80.1 ± 0.6% cells in case of AmB-NA3 and AmB-NA5 respectively. AmBisome maintained cell viability up to 84.5 ± 2.7%; while pure AmB as well as Fungizone was 100% lethal at the same concentration (200 μg/mL AmB equivalents). MTT assay performed on J774A.1 cells treated with AmB-NA showed similar results. The cell viability was comparable to AmBisome with much higher survival rate (~90% at 200 μg/mL AmB equivalents) than that observed with HEK-293 cells (Fig. 4C). The morphology of J774A.1 cells was well preserved when cells were incubated in the presence of varying concentration of AmB-NA5 (Fig. 4D(i)–(iv)). Even at high AmB concentration (100 and 200 μg/mL), insignificant toxic effects were seen (Fig. 4D(iii) and D(iv) respectively) as most of the cells retained normal cellular organization. LDH release assay is generally used as a tool to study cell lysis. We employed the same assay to determine AmB induced lysis of target cells (Fig. 4E). At 100 μg/mL, Fungizone and free AmB caused 100% LDH release, followed by AmB-NA3 (24.2%), AmBisome (14.6) and AmB-NA5 (14.5%) formulations.

**Table 2:** *In vitro* and *in vivo* toxicity of amphotericin B formulations.

AmB Formulation	IC_50_ (mg/L of amphotericin B) against mammalian cell line^a^		MTD (mg/kg of amphotericin B)^b^
	HEK-293 cell line	J774A.1 cell line	
Fungizone	26.5	22.5	2
AmBisome	201	>200	>15
AmB-NA3	120	>200	15
AmB-NA5	176	>200	15

^a^ *In vitro* toxicity of different formulations of amphotericin B towards mammalian (J774A.1 and HEK-293) cell lines after 24 h of incubation (IC_50_) using the MTT test.

^b^ *In vivo* toxicity (Maximum tolerated dose, MTD) of different formulations of amphotericin B in Balb/c female mice after a single bolus injection; values are calculated from the number of mice surviving the injection.

### *In vivo* toxicity assays

*In vivo* toxicity data (Table 3) suggest that in contrast to toxic Fungizone, AmB-NA did not affect liver and kidney functions of the host adversely. There was no statistically significant difference (p > 0.05) in the levels of transaminases (ALT and AST), between the AmB-NA-treated mice and those of the control healthy group. Moreover, at lower concentrations, animals treated with both AmB-NA formulations showed normal levels of the kidney function parameters. However, elevation in kidney and liver function markers was observed at a dose of 15 mg/kg body weight for both the nano-formulations. AmBisome as well as both the AmB-NA formulations were well tolerated by experimental animals upto a dose of 10 mg/kg body weight. The treated mice did not show any sign of illness or loss in body weight (data not shown). On the contrary, mice injected with Fungizone had significantly increased BUN (blood urea nitrogen) and serum creatinine concentrations (Table 3).

**Table 3:** Effect of amphotericin B formulations on hepatic and renal parameters.

Amphotericin B Formulations	Dose (mg/kg)	ALT^a^ (IU/L)	AST^a^ (IU/L)	BUN^a^ (mmol/L)	Creatinine (μmol/L)
Control^b^	0	149.8 ± 14.8	18.0 ± 2.0	5.4 ± 2.6	35.5 ± 1.02
AmB-NA3	1	148.3 ± 22.8	19.3 ± 1.9	5.8 ± 0.3	37.5 ± 0.06
	5	161.2 ± 8.4	17.3 ± 1.7	6.2 ± 1.9	39.3 ± 1.71
	10	154.0 ± 29.4	20.6 ± 7.0	5.5 ± 2.6	42.5 ± 1.00
	15	350.0 ± 2.4*	31.8 ± 5.7*	8.8 ± 2.3*	58.5 ± 1.49*
AmB-NA5	1	146.3 ± 2.8	17.3 ± 1.1	5.8 ± 1.3	39.5 ± 1.60
	5	162.8 ± 19.2	18.8 ± 4.5	6.0 ± 1.2	36.6 ± 1.52
	10	167.1 ± 8.4	19.3 ± 2.7	6.2 ± 1.9	40.0 ± 1.87
	15	335.0 ± 8.4*	27.8 ± 2.4*	8.3 ± 1.3*	56.5 ± 1.91*
Fungizone	2	704.0 ± 27.6*	49.0 ± 2.2*	16.2 ± 0.2*	88.7 ± 2.22*
AmBisome	10	205.2 ± 14.3*	24.8 ± 3.8*	5.7 ± 1.0	46.3 ± 3.14

The two commercial formulations of Amphotericin B, Fungizone and AmBisome, were used at dose of 2 and 10 mg/kg, respectively.The new formulations (AmB-NA3 and AmB-NA5) were used in a range of concentrations (1–15 mg/kg). Values are mean ± SD.

*Significant at *P* < 0.05compared with the results for the respective control mice, Student’s t test.

^a^ALT, alanine aminotransferase; AST, aspartate aminotransferase; BUN, blood urea nitrogen.

^b^Control mice were given only PBS.

Various hematological parameters like RBC counts, hemoglobin content, percent hematocrit, and platelet count were evaluated in a mouse model after exposure to AmB equivalents of 1, 5 and 10 mg/kg/day (Table 4). The results indicate that there was significant difference (p > 0.05) in all hematological parameters for Fungizone as compared to control healthy group. Both AmB-NA and AmBisome formulations did not induce significant decrease (p > 0.05) in any of these parameters except platelet count.

**Table 4:** Hematological parameters on post-injection effects at day 8 after seven doses (1, 5, and 10 mg/kg/day of AmB).

Parameters^a^	Dose (mg/kg/day)	Fungizone	AmBisome	AmB-NA3	AmB-NA5	Control^c^
RBC^b^ (×10^6^/mL)	1	4.03 ± 0.4*	7.38 ± 1.8	7.45 ± 0.9	7.31 ± 1.3	7.47 ± 0.2
	5	—	6.58 ± 0.7	7.01 ± 1.1	7.06 ± 1.6	
	10	—	4.41 ± 1.7	7.00 ± 0.6	6.90 ± 0.2	
WBCs^b^ (×10^3^/μL)	1	4.17 ± 0.6*	6.20 ± 0.8	6.20 ± 1.5	6.40 ± 2.2	6.7 ± 0.4
	5	—	6.00 ± 0.6	6.10 ± 0.4	6.20 ± 0.1	
	10	—	5.7 ± 0.9	5.80 ± 0.8	6.00 ± 1.2	
Platelet (×10^3^/μL)	1	155 ± 21*	460 ± 24	400 ± 35	442 ± 45	507 ± 24
	5	—	410 ± 11	305 ± 16	398 ± 23	
	10	—	342 ± 32*	220 ± 22*	312 ± 12*	
Hematocrit (%)	1	24.4 ± 2.9*	40.3 ± 0.5	41.6 ± 1.2	44.6 ± 2.1	46.2 ± 2.4
	5	—	35.5 ± 2.0	41.0 ± 0.6	43.2 ± 2.8	
	10	—	36.5 ± 2.8	39.1 ± 2.9	42.0 ± 1.2	
Hemoglobin (g/dL)	1	11.5 ± 1.1*	16.4 ± 0.9	16.9 ± 1.2	17.1 ± 0.4	17.2 ± 1.2
	5	—	16.0 ± 0.3	16.5 ± 0.7	16.8 ± 0.9	
	10	—	15.3 ± 2.2	16.2 ± 1.1	16.5 ± 1.4	

The commercial formulation of Amphotericin B, Fungizone was given at dose of 1 mg/kg for 7 days.

^a^All parameters were expressed as mean values ± SD.

^b^RBC, red blood cells; WBC, white blood cells.

^c^Control groups received only PBS.

*Significant at *P* < 0.05, Students t test.

### *In vitro* stability studies

The AmB-NAs were found to be stable upon storage at 4 °C, exhibiting little loss of AmB content (5% loss) and insignificant change in size upon incubation for up to 30 days (Table 5). The AmB-NA5 samples stored as lyophilized powder (Table 5) or suspension (Table S2) were stable at 37 °C, as more than 92% of the AmB remained associated with AmB-NAs after 30 days. The AmB-NA5 did not show any instability when subjected to freeze thaw cycles. There was insignificant increase in size upon freeze thawing (Table S3).

**Table 5:** Stability studies of freeze-dried AmB-nanoassembly stored as powder.

Characteristics	0 days	7 days		15 days		21 days		30 days	
		4 °C ± 1 °C	37 °C ± 2 °C	4 °C ± 1 °C	37 °C ± 2 °C	4 °C ± 1 °C	37 °C ± 2 °C	4 °C ± 1 °C	37 °C ± 2 °C
% AmB content	100	99.5 ± 0.5	99.2 ± 1.4	98.7 ± 2.7	98.9 ± 3.9	97.5 ± 3.2	96.8 ± 2.9	95.4 ± 1.1	92.2 ± 1.2
Size distribution (nm)	102 ± 9	94 ± 2	104 ± 4	104 ± 5	109 ± 2	103 ± 7	106 ± 1	106 ± 6	107 ± 7

### MTD in healthy mice

The MTDs of Fungizone and AmB-NA, determined at 24 hr, post single-dose intra-venous treatment in the mice, are presented in Table 2. Although, the literature suggests 2.0 mg/kg MTD for Fungizone^40^, however, all the treated mice died within 30–120 min after administration of same dose of Fungizone, probably due to pulmonary embolism. In contrast, AmB-NAs induced a delayed mortality at the highest doses tested for both AmB-NA3 and AmB-NA5, the MTD was 10 mg/kg; further increase of the dose resulted in impaired kidney function, whereas mortality of mice was seen at dose of 15 mg/kg.

### *In vitro* antifungal activity

**Figure 5:**
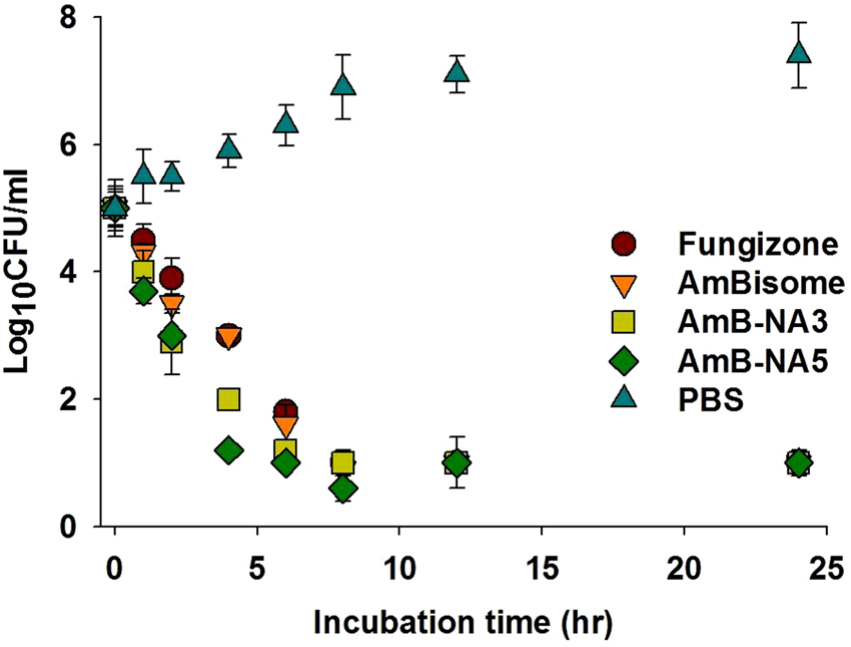
AmB-NA formulation demonstrated strong antifungal activity against fungal pathogen: (**A**) Representative time–kill curve plot for *C*. *albicans* in the presence of Fungizone, AmBisome or AmB-NA at 4 × MIC. Wells containing no antibiotic were taken as control. Assays were performed in triplicate. Each result is representative of at least three separate experiments. Values represent mean ± SD.

The MIC of as-synthesized AmB-NAs against *Candida spp*. was 0.125–0.5 mg/L and 0.125–0.25 mg/L for AmB-NA3 and AmB-NA5, respectively. The MIC of Fungizone against *C*. *albicans* isolate was found to be 0.25–1.0 mg/L (Table 6). Fungicidal activity is routinely defined as a 99.9% reduction in CFU over a fixed sampling period. MFC values were found to be 0.25–0.5 mg/L for both AmB-NAs, while Fungizone showed MFC of 0.5–2.0 mg/L. MIC and MFC against *Aspergillus fumigatus* was found to be 1 and 8 mg/L respectively. When different standard AmB formulations were tested at 4 × MIC, AmB-NA as well as Fungizone caused a 99% loss of yeast viability after 4 h of incubation (Fig. 5).

**Table 6:** *In vitro* antifungal activity of various AmB formulations.

Strains	AmB-NA3		AmB-NA5		AmBisome		Fungizone	
	MIC (mg/L)	MFC (mg/L)	MIC (mg/L)	MFC (mg/L)	MIC (mg/L)	MFC (mg/L)	MIC (mg/L)	MFC (mg/L)
*C*. *albicans* ATCC 90028	0.5	0.5	0.25	0.25	0.5	0.5	1	1
*C*. *albicans* ATCC 18804	0.25	0.5	0.25	0.5	0.5	1	0.25	0.5
*C*. *albicans* ATCC 10231	0.25	0.25	0.25	0.25	0.5	1	0.25	0.5
*C*. *glabrata* 4224 A	0.125	0.25	0.125	0.25	0.125	0.25	0.25	1
*C*. *glabrata* MTCC 3019	0.25	0.5	0.25	0.5	0.5	1	1	2
*A*. *fumigatus* ATCC 9157	1	8	1	8	4	16	1	8

### Antifungal efficacy of AmB NA against experimental murine candidiasis

**Figure 6:**
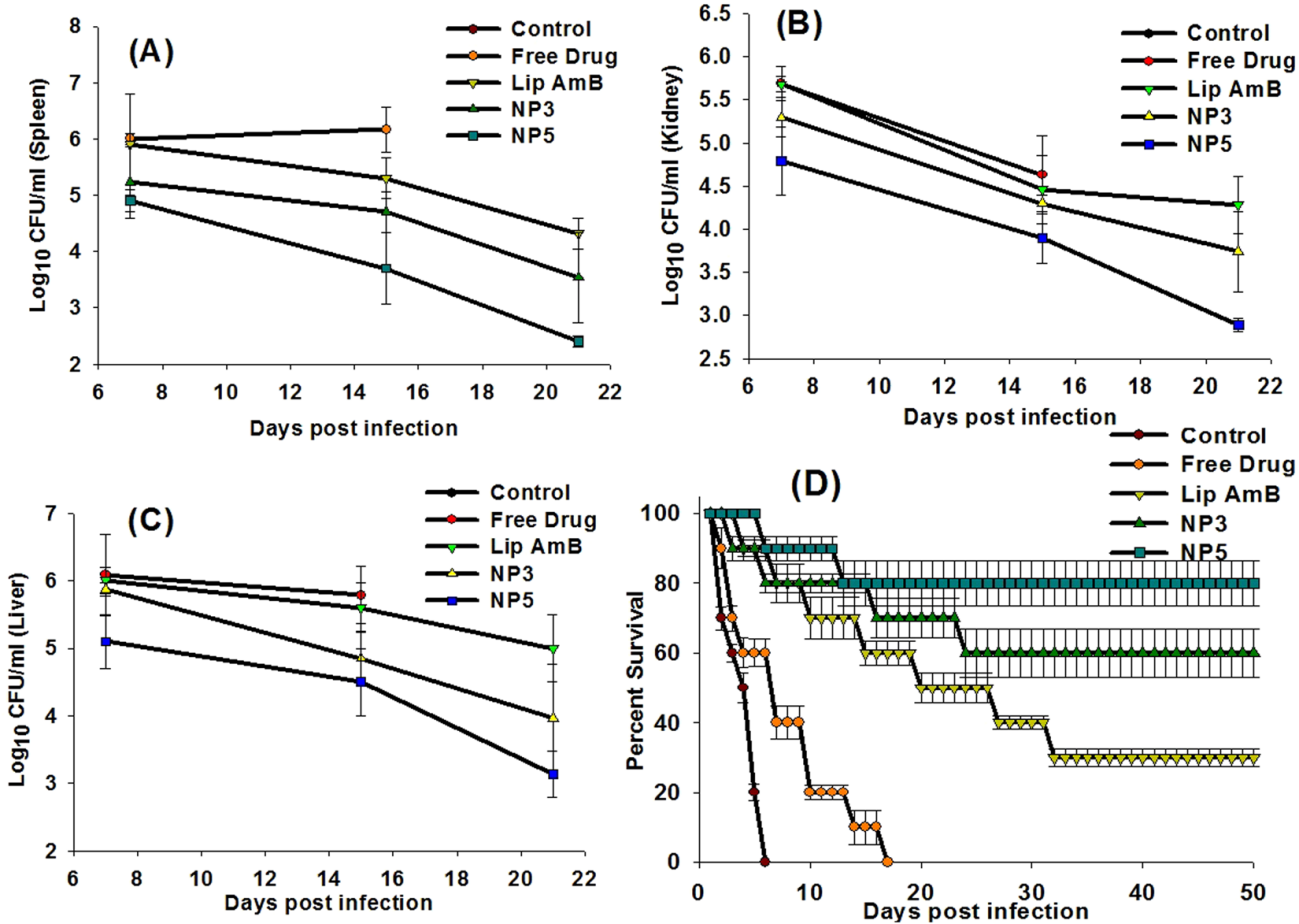
Potential of AmB-NA in treatment of systemic *Candida albicans* infection in experimental animals. Residual fungal load in the vital organs of experimental animals after treatment with AmB-NA formulation. (**A**) Fungal load in spleen. (**B**) Fungal load in Kidney (**C**) Fungal load in liver. [Group I versus Group III (p value < 0.001); Group I versus Group IV (p value < 0.001); Group I versus Group V (p value < 0.001); Group III (Lip-AmB) versus Group V (AmB-NA5) (p value < 0.05)]. (**D**) Survival rate of animals after exposure to infection followed by treatment with AmB-NAs. Group I versus Group III P value < 0.001; Group I versus Group IV P value < 0.001; Group I versus Group V P value < 0.001; Group III (Lip-AmB) versus Group V (AmB-NA5) P value < 0.05.

As shown in Fig. 6, there was a significantly higher survival rate (80%) in group of animals that was treated with AmB-NA5 (Group V), with respect to 60% survival in the group of animals (Group IV) treated with AmB-NA3 (p value < 0.05). Animals treated with PBS only (Group I) died within 5 days post infection, while those treated with the free form of AmB at the dose of 5 mg/kg body weight (b.w.) (Group II) did not survive beyond day 14 post-infection (p value < 0.01). Substantiating survival data, there was a significant decrease in fungal load in the vital organs of the animals of Groups IV and V (AmB-NAs) at days 7, 15 and 21 post-infection (p value < 0.001). AmB-NA5 formulation was effective in reducing the fungal burden in the liver and spleen to greater extent, while animals treated with AmB-NA3 also exhibited considerable reduction in fungal load by 15 days post-infection (Fig. 6A,B and C), although it was less significant than that of AmB-NA5 (p value < 0.01). The free drug, (Group II), was unable to show any notable reduction in *C*. *albicans* count from the vital organs of the infected animals. Control animals were not available for fungal burden data as they did not survive beyond day 7 post-infection.

## Discussion

Earlier attempts to formulate novel drug delivery system for AmB with better therapeutic index^41–43^ had met with little success. In general, low drug loading^42^, excipient related toxicity issues^44^, and unfavorable physicochemical properties^45^ were cited as related drawbacks. Some polymeric systems suffer from instability in serum^46^, resulting in sudden or burst release of the payload. AmBisome remains the most efficient formulation in terms of usage spectrum, pharmaco-kinetics and low toxicity issues^47^. Nevertheless, issues like high price due to the cost of phospholipids^7,8^, complex production process^9^ and critical loss of AmB activity^10–12^ pose limitations for its wide usage across the world, especially in the third world countries. Moreover, extremely rare reports of AmBisome related severe hypersensitivity reactions or pseudo-hyperphosphatemia have also been documented^48,49^. Such developments certainly warrant proper support and close supervision when AmBisome is used for treatment.

It has been shown that the aggregation of AmB directly affects its cytotoxicity against host cells^13,14^ as well as its antimicrobial activity against fungal pathogens^20,50^. Earlier report involving heat induced AmB poly-aggregates, demonstrated diminished toxicity both *in vitro* and *in vivo* ^18–20^, than the small soluble water aggregates (oligomers) present in Fungizone. Unheated Fungizone was toxic at 1 mg/kg, while it was possible to inject safely 2.5 mg of the heated formulation per kg b.w.^17^. The reduction in toxicity had already been demonstrated for both healthy mice and for mice with candidiasis or cryptococcosis^51^. The super-aggregates of AmB exhibited only a slight reduction of *in vivo* activity against *Candida albicans* ^19^ but an increase in its activity against *Leishmania donovani* ^17^. It can, therefore, be speculated that AmB toxicity can be reduced by designing a novel derivative/formulation that shows higher degree of drug aggregation.

Our earlier efforts to exploit the potential of *Aloe vera* leaf extract in inducing bioformation of nucleobase assemblage have been quite encouraging^36^. This led us to hypothesize that *Aloe vera* extract can be used to induce synthesis of other drug entities including AmB nano-structures. The biomimetic synthesis could have great implication as nanoassembled form exhibit superior properties as compared to the pure drug^22^ (referred to here as free form of the drug). We incubated AmB with *Aloe vera* extract maintaining similar conditions as that of our previous study^36^. Initially, spectroscopic studies were performed to monitor the formation of AmB nano-structures. The as-synthesized nano-structures demonstrated spectra typical of AmB super-aggregates. The AmB-nano-assemblies were then further characterized and evaluated for their toxicity and activity.

The present study describes *Aloe vera* leaf extract mediated transformation of AmB into a super-aggregated form, which can act as a potential reservoir to facilitate sustained release of parent AmB molecules. The biomimetic synthesis of AmB super-aggregates was evident from absorption spectrum showing hypsochromic shift in the λ_max_. With time, the peak at short-wavelength undergoes a further blue shift that gets stabilized at 340 nm after completion of the reaction (24 h) (Fig. 1A). Nano-assemblies precipitated by centrifugation were subjected to several washing steps in order to eliminate any possibility of the presence of *Aloe vera* extract. The washing process did not induce any significant alteration on spectral properties as absorption spectra before and after washing remain unchanged (data not shown).

The spectral modifications induced by aggregation may be represented by the ratio of the first (340 nm) to the fourth (410 nm) peaks, I/IV (A_340_/A_410_)^13^, indicative of the aggregated versus monomeric ratio. In accordance with previous reports^42,52^, the A_340_/A_410_ ratio was >2 for AmB-NA, indicating the presence of super-aggregated forms^13^ of AmB in AmB-NA. Absorption peaks at a lower wavelength are generally observed when aggregated AmB is complexed with deoxycholate^53^, or upon formation of superaggregates by heat treatment of AmB alone^15^. It has also been shown that AmB exists in aggregated form in AmBisome; exhibiting hypsochromic shift of 7–9 nm in λ_max_^54^. This shift could be accredited to the close association of AmB to the lipid bilayer^55^.

When excited at 325 nm, the florescence spectra of AmB-NA displayed hypsochromic shift; suggesting the biomimetic synthesis of AmB super-aggregates (Fig. 1B). The observed shift can be attributed to the presence of self-associated AmB species^37^. It can be inferred that the fluorophores of AmB are experiencing relatively less polar environment upon nano-assembly formation. This situation is possible when AmB molecules are stacked upon each other, thereby leading to the formation of compact particulate nanoassemblies. The AmB super-aggregation leads to the formation of precise nanostructures with characteristic spectral properties. XRD and FTIR data (Figs 1C and 2D) suggest that the assemblage of drug molecules in nanoassemblies does not alter its basic chemical entity. It can be inferred that the parent structure of AmB molecule is retained during synthesis of AmB-NA.

In aqueous dispersions, AmB has a tendency to exist in three different forms viz. monomer, soluble and insoluble aggregated states^15^. In a system where AmB exists in monomeric form (organic solvents such as DMSO), a well-defined electronic absorption spectrum in the range between 300 and 450 nm (λ_max_ at ~405–409 nm) appears^13,53^; which can be correlated with the π-π* transition in the chromophore subunit^56^. In contrast, when AmB is solubilised in a small amount of DMSO and diluted with water, it shows a spectrum of broad band at ~328–340 nm depicting minimal peak at 410 nm; characteristic of highly self-aggregated form^15^.

The UV absorption spectra were recorded both for aqueous suspension of AmB-NAs as well as its free form (Figure S1). For monomeric form, the main absorption band, with the 0–0 vibrational maximum ca 408 nm, corresponds to the strongly permissible electronic transition from the ground energy level S_0_ (1^1^A_g_) to the so-called excited S_2_ energy level (1^1^B_u_)^37,57^. In the aggregated form, absorption band at 408 nm is less prominent. This behavior of AmB can be explained on the basis of excitonic band splitting model^58^, which suggests that the direct electronic transition from the S_0_ (1^1^A_g_) to the energy level S_1_ (2^1^A_g_) is optically forbidden due to the symmetry reasons. The fluorescence emission spectrum, recorded with the excitation in the 0–0 transition region, presents two distinct bands corresponding to de-excitation of the S_2_ and the S_1_ energy states: the direct emission and also the emission from the S_1_ state after the non-radiative S_2_ → S_1_ relaxation. Fluorescence emission from both the energy levels is typical for polyenes characterized by the conjugated double bond system n = 7^59^; although this happens only in case of monomeric form. For aggregated structures, fluorescence emission originates from the excitonic band located below the S_1_ level on the energy scale. Additionally, emission band, assigned to the S_1_ → S_0_ transition is not homogeneous, either due to possible overlap of various electronic energy levels of monomeric form or due to the presence of different molecular forms of AmB existing in the sample. Thus, the extent of excitonic band splitting has been shown to increase with the extent of aggregation^37^.

The two forms of AmB with characteristic spectrum are now interpreted as monomeric and aggregated states^60^ with the chromophore-chromophore distance calculated to be 4.8 Å. This implies relatively tight packing of nonpolar polyene chains; with neighboring interacting molecules consisting of roughly equiprobable population of parallel or antiparallel AmB dimers that rapidly interconvert into each other^61^. In Fungizone, the molecular assembly is constituted out of the dimeric subunits. Each dimer is twisted by ca. 170° and stabilized via the dipole-dipole interactions between chromophores and additionally, via hydrogen bonds, between the hydroxyl groups C_35_-OH and oxygen C_42_-O-C_19_ of the two monomers. Molecular modeling studies showed AmB dimers can form tetrameric structures, in which two AmB dimers are linked via the hydrogen bond C_8_-OH···OH-C_3_ that can easily associate into tetrameric structures. The resulting molecular aggregate of AmB (tetramer) adopts a pore-like structure with roughly elliptical cross-section (4.0 × 7.5 Å) that span lipid bilayers and act as a trans-membrane ion channel^60^; contributing to the toxicity towards cholesterol-rich membranes. The solvent-exposed hydrophobic surface of dimers and tetramers (ca. 900 Å^2^) is larger than that of a monomer (ca. 650 Å^2^), indicating again that dimers and tetramers can have greater affinity for membrane insertion than monomers^61^.

As per classical nucleation theory proposed by Lamer^62^, the formation of primary particles is described as a self-nucleation process at the initial stage. The formed nuclei then act as seeds for the particle growth leading to the coalescence of the nuclei, accompanied by an increase in the thermodynamic stability. Afterwards, the formation of nanoparticles takes place by means of heterogeneous nucleation and growth; a process referred to as Ostwald ripening^22^. Molecular modeling studies demonstrated that AmB tetramers become more stable during the course of the simulation, retaining large hydrophobic solvent-accessible surface area (SASA) upon association. This suggests that higher oligomers trap the monomers in their initial orientation and inhibit structural rearrangements^61^. It can, therefore, be stipulated that the AmB dimers/tetramers can act as seeding point (nucleus) for the condensation of monomers and eventually lead to the formation of super-aggregated NAs. The contents of *Aloe vera* leaf extract function both as a driving force as well as stabilizing agent for the whole process.

According to Chou and Chen^63^, several parameters such as temperature, concentration of parent molecule, incubation time *etc*. play an important role in regulating the size of the as- formed nanoparticles. We explored AmB NA fabrication by manipulating AmB concentrations, incubation time and amount of *Aloe vera* leaf extract. In *Aloe vera* leaf induced nanoparticle fabrication, the size of AmB-NAs first reduces followed by increase with increment in the concentration of AmB; size range of the order of 100 nm was obtained while using 1 mM AmB concentration (Fig. 2E (i)). Although the reduction in the size is small (i.e. from 132–102 nm), the reason behind this kind of behavior is not yet known. It was previously shown that the size of heat-induced super-aggregates increases with increasing AmB concentration, probably due to the rapid agglomeration of AmB molecules at 70 °C; leading to much greater size dimensions (~300 nm)^20^. In the present study where the nanoparticles are obtained by treatment with *Aloe vera* leaf extract, the size distribution seems to be regulated by the slow reaction rate as well as the presence of reducing agents.

Earlier, it has been reported that the shape and size of nanoparticles can be controlled based on the volume of *Aloe vera* leaf extract^30^, with higher amount of extract leading to an increase in the population of the spherical particles. We observed that higher amount of *Aloe vera* leaf extract ensued in increase in the size of NAs (Fig. 2E(ii)). It can be stipulated that larger volumes of *Aloe vera* leaf extract may provide a more favourable environment, leading to more of nucleation and condensation reactions; thereby facilitating the formation of larger nanoassemblies. Time kinetics revealed a general increase in size of the NAs upon increase in incubation time (Fig. 2E(iii)). With time, the nucleation-condensation reactions may occur in bulk, leading to the formation of larger nano-aggregates. AmB NAs formed using 2 mM AmB concentration were found to be toxic in subsequent studies, while those prepared with 0.5 mM AmB concentration exhibited stability issues (data not shown). However, 1 mM AmB concentration displayed optimum formation of nano-aggregates with low toxicity and high stability, and was used in subsequent studies.

TEM analysis confirmed the presence of nearly spherical structures with a mean particle size of ~65 nm (Fig. 2A). The size of nanoparticles when measured by DLS used to be always larger (~105 nm) than the particle size determined employing TEM analysis. In fact, different methods of size-determination can produce conflicting data. TEM provides information about the size and shape of individual nanoparticles dried on a substrate under high vacuum^64^. Dynamic light scattering (DLS) measures diffusion in particle dispersions, which can be interpreted using the Stokes-Einstein equation to yield an ensemble average hydrodynamic particle diameter^64^. Hence, there is often a discrepancy between TEM and DLS based size determination.

We observed that treatment of *Aloe vera* leaf extract with Proteinase K, DNase or RNase does not alter its ability to induce formation of AmB-NAs (Figure S2A). This observation rules out any possibility of involvement of nucleic acid or protein based factors present in *Aloe vera* leaf extract in the synthesis of AmB-NAs. Further, it was found that boiling of extract for 15 min does not eliminate its nano-assembly synthesis potential (data not shown). The *Aloe vera* leaf extract was then fractionated into two different parts, using a 2 kDa nylon dialysis bag. The fraction with molecular weight less than 2 kDa was able to induce formation of NAs (Figure S2B), while the fraction with molecular weight (MW) greater than 2 kDa failed to induce AmB-NA synthesis. This suggests that low molecular weight components of *Aloe vera* leaf extract are instrumental in fabrication of AmB-NA^30^. It seems that the slow reaction rate of particle formation is induced by shape-directing ability of the carbonyl compounds present in the *Aloe vera* leaf extract^30^. In fact, various plant metabolites, including terpenoids, polyphenols, anthraquinones, alkaloids, phenolic acids, proteins and sugars have been considered responsible for bio-reduction of metal ions, yielding nanoparticles^22^^.^

It is stipulated that anthraquinones, with excellent redox properties, could act as electron shuttle^65^ in the reduction of metal-based nanoparticles. Anthraquinones such as Aloin, Emodin, Cyperquinone, Remerin *etc* undergo keto-enol tautomerism, where the enol form acts as the reducing agent. To test this hypothesis, we performed GC-MS (Gas Chromatography-Mass spectrometry) analysis of *Aloe vera* leaf extract, and found various peaks corresponding to small sized plant metabolites, that are previously reported to be present in *Aloe vera* ^66^. A recent study reports that Aloin actively induces the nanofabrication of precursor chemical molecules^67^. Another study demonstrated that aloin and emodin are responsible for the synthesis of metal based nanoparticles^68^. Our GC-MS data clearly showed the presence of these components in *Aloe vera* leaf extract, and suggest their possible role in induction of nano-assemblies. To further ensure this, we procured commercially available pure Aloin(Sigma-Aldrich, USA) and tested its potential to induce nanoparticle production. We found that aloin, infact, readily facilitates the nanoassembly of AmB (Figure S2C). However, the size of the nanoparticles was much smaller as compared to the size of nanoparticles synthesized using the *Aloe vera* whole leaf extract (Figure S2D). It can be speculated that in a manner similar to that of metal nanoparticle fabrication, anthraquinone components of *Aloe vera* leaf extract induce AmB-NA fabrication.

The as-synthesized AmB nano-formulation was assessed for its degradability under simulated physiological conditions. The release pattern of the AmB−NA in PBS (Fig. 3A) was comparable to that of AmBisome^9^. The biphasic pattern of drug release observed is analogous to that reported for other lipidic and polymeric carrier based reservoirs of AmB^69,70^. Since *in-vivo* stability in biological fluid is an important criterion for its future use, change in AmB-NA drug content in the presence of serum was monitored over the time. The AmB-NA showed high plasma stability with sustained AmB release (Fig. 3B). In contrast, some of the extensively studied systems including those derived from polymers demonstrated reduced stability in serum^71,72^. Most of the earlier studies to examine the stability of the polymeric micelle system were based on PBS, while no data on release of AmB in serum was shown^71,72^. Additionally, in order to mimic the intestinal absorption, release kinetics in Histidine buffer was performed. Minimal drug release (less than 30%) was observed in Histidine buffer (Fig. 3C). The AmB release profile was analyzed using various kinetic models to know the release mechanism. AmB release from Fungizone is believed to occur by diffusion mechanism as it has a matrix type system (micellar formulation). Fungizone follows release kinetics as per Korsmeyer-Peppas and the Higuchi model; while the release from AmBisome follows zero order fashion^73^. The drug release from AmB-NA occurs by a diffusion-controlled process described by the Higuchi model as well as the Hixon Crowell model.(Table S4). Higuchi model is generally applied to diffusion of solid drugs dispersed in homogenous uniform matrix. The Hixson-Crowell model applies to pharmaceutical dosage forms such as tablets, where the geometrical shape of the pharmaceutical dosage form diminishes proportionally over time.

Previous toxicity studies have shown that at low concentrations (<2 µM), AmB induces a reversible membrane permeabilizing effect, while at higher concentrations it perturbs plasma membrane of RBC leading to its lysis^14^. The AmB concentration required to trigger 50% K^+^ leakage from RBCs is much higher in case of AmB-NA3 or AmB-NA5 when compared to free form of the drug (Fig. 4B). There was 50% haemolysis after 24-h incubation of Fungizone solutions (~7 µg/mL), while AmB-NA-treated RBCs remained intact even at 50 µg/mL AmB equivalents (Fig. 4A). This is consistent with the findings of Gaboriau *et al*.^19^ who reported decreased haemolysis of red blood cells by HT-AmB (super-aggregated AmB prepared by heat treatment) as compared with Fungizone. The investigators also reported reduced toxicity to the HT29 cell line as measured by lactate dehydrogenase release and the MTT assay^19^. Similarly, studies performed by Rogers *et al*.^74^ on a human monocytic cell line (THP-1) showed no effect on cell viability for superaggregated-AmB; while fungizone elicited dose-dependent toxicity. We found that AmB-NAs exhibit several fold less toxicity against the mammalian cell lines (HEK-293 and J774A.1) as compared to Fungizone (Fig. 4C and D). The *in-vivo* toxicity studies using mice showed that the animals could tolerate relatively high dose (10 mg/kg b.w.) of AmB-NA (Table 3). Slight kidney and liver toxicity was observed at much higher dosages (15 mg/kg). Thus, AmB-NA exhibits better protection from the acute toxic effects seen with conventional Fungizone formulation.

In our study, the *Aloe vera* leaf extract contents used in the preparation of the AmB nano-assemblies were removed by extensive washing of the nanoparticles with water. Nevertheless, we tested the toxicity of *Aloe vera* extract on RBCs. The extract did not induce any significant toxicity to RBCs at the highest concentration tested. Less than 5% of RBC lysis was found when ~250 μL of *Aloe vera* extract (at a concentration of 5%) was used (Figure S3). Several studies have determined the toxicity profile of the *Aloe vera* extract. In one study, the lethal dose (LD_50_) in Swiss albino mice was found to be 120.65 mg/kg^75^. Another acute toxicity study demonstrated a maximum tolerated dose of 100 mg/kg body weight and LD_50_ of 250 mg/kg^75^. The extensive washing of as-synthesized NA ensured that even traces of *Aloe vera* extract were removed.

Considering shelf life as an important feature of any formulation before it reaches the common user in the market, we performed stability tests for AmB-NA formulations (Tables 3 and S2). At the normal storage condition i.e. 37 °C ± 2 °C, AmB-NA5 underwent negligible change in size and drug release. After 4 weeks, drug load in the AmB-NA was found to be 92% of the initial content. The freeze-dried product showed no significant change in drug content and particle size at 4 °C (Table 3). The product had an AmB content of 95% of the initial amount as determined by HPLC at the end of 4 weeks. There was no apparent difference in physical characteristics such as colour, odour *etc* of the as-synthesized ﻿AmB-NA f﻿ormulation. It was mandatory to ensure that the low toxicity of AmB-NA could be maintained after storage. The storage of the freeze-dried powder at 4 °C for 30 days did not induce RBC haemolysis (Figure S4A). This observation validates cold chain stability of AmB-NAs. AmB-NA5 stored for 4 weeks at 37 °C as non-reconstituted solid did not cause RBC haemolysis after a 24 h incubation period (Figure S4B). Importantly, when AmB-NA5 was stored for 7 days as a sterile solution (1 mg/mL AmB equivalent) at 37 °C, it also did not display detrimental effects on RBCs (Figure S4C). This suggests that AmB does not readily leach out or disassemble from the nanoparticles on storage. Furthermore, the stability of the treated AmB solution (super-aggregates) was shown to be higher than that of the untreated ones (free AmB). There was almost no change in the total content of the AmB super-aggregate solutions (at 1 mg/mL AmB equivalent) when different formulations were incubated with stirring for 1 day at 37 °C (data not shown).

In general, super-aggregates (heat-treated AmB) are more stable compared to the water soluble aggregates (unheated AmB)^19^. On the other hand, Fungizone^®^ should not be stored for more than 8 hours at room temperature (25 °C) or 24 hours in a refrigerator (2–8 °C), and the in-use storage time of AmBisome^®^ would normally not be longer than 24 h at 2–8 °C^76^. Moreover, autoxidation of Fungizone solutions leads to a 20% decrease in the total AmB content after 1 h incubation^19^. AmBisome has been shown to undergo a rapid increase in particle size as well as radical change in drug content upon storage^71^. The apparent high stability of the AmB-NA in solution could be due to the small size and negative charge distribution (−34 mV, and ∼100 nm) (Table 1). The charge-charge repulsion between similar charged nanoparticles prevents aggregation of the particles and maintains a stable system^77^. The elevated stability profile of AmB-NA could also be attributed to the high chemical stability of the AmB super-aggregates, which are less susceptible to peroxidative process than its soluble aggregated form (oligomers) and, therefore, had less affinity to cholesterol harbouring membranes^37,57^. The super aggregates help in maintaining the concentration of AmB below its critical aggregation concentration (~10^−6^ M). As a result, the drug remains in its monomeric form, thereby rendering AmB-NA safer. It has been suggested that AmB monomers permeabilize only ergosterol-containing membranes, while the side effects are due to the interactions of self-associated forms, possibly dimers/oligomers, with cholesterol-rich membranes^61^.

The desirable pharmacokinetics features of AmB super-aggregate led us to further assess their *in-vitro* activity against various opportunistic fungal pathogens including *Candida* spp. The nanoparticles based formulation was found to be efficacious against *C*. *albicans*. The AmB-NA formulation was also effective in killing other related *Candida* spp. and *Aspergillus fumigatus* with equal propensity. The lower MIC and MFC values of AmB-NA established their future utility against important fungal pathogens (Table 6). Further evaluation by time-kill study showed that in the presence of the complexes at concentrations of 4 × MIC, the CFU/mL rapidly decreased. Between 0–4 h post treatment, the reduction was more than 4 log CFU (Fig. 6A,B and C). This level of suppression was maintained throughout the monitoring process and suggests that the activity was fungicidal rather than fungistatic. The antifungal efficacy of AmB-NA was also evaluated in experimental murine candidiasis in Balb/C mice. The greater animal survival as well as lower residual fungal burden in various vital organs data suggests that AmB-NA showed higher efficacy at par to that of well-established AmBisome against *C*. *albicans* infection (Fig. 6D). Similar activity profile has been reported for AmB super-aggregates prepared by mild heating (hAMB-DOC) against *Leishmania donovani* ^17^ and *Plasmodium falciparum* ^78^. Improved therapeutic efficacy of hAMB-DOC was also demonstrated, compared to efficacy of conventional Fungizone, in a model of invasive candidiasis in immunocompetent mice^51^. In persistently leukopenic mice with severe invasive candidiasis, higher dosages of hAMB-DOC were tolerated than those of conventional Fungizone (3 versus 0.8 mg/kg of b.w. respectively), resulting in significantly improved therapeutic efficacy^20^.

The mechanism by which the AmB-NA complexes exert their greater antifungal efficacy is not known. AmB-NA being particulate in nature, can be avidly phagocytosed by the macrophages. It has been reported that the nano-particle bearing macrophages may act as ‘Trojan horses’^79^ that can carry the engulfed nanoparticles. Since macrophages are involved in the pathogenesis of candidiasis, they can act as ‘secondary depot’^80^, or ‘cellular drug reservoirs’, gradually releasing the drug into the surrounding milieu^81^. Thus, they may provide an indirect boost to microbial clearance as the drug can be delivered to the site of active pathogen growth^82^. This may lead to greater distribution of AmB in organs of reticuloendothelial system (RES) to liver and spleen, thereby effectively reducing the pathogen burden. Our pilot studies revealed massive deposition of AmB in reticuloendothelial organs (data not shown). Moreover, biodistribution studies have revealed intense accumulation of Amphocil^®^ (amphotericin B colloidal dispersion) and Abelcet^®^ (amphotericin B phospholipid complex) in the mononuclear phagocyte system^83^. Infact, RES constitutes a major pathway for the accumulation of poly-aggregated forms of AmB^54,84^. Although initially demonstrated in cancer, this paradigm may be extended to other nano-therapeutics and non-cancer disease indications^85^ in which macrophages accumulate near the target tissue. It seems, therefore, plausible that AmB-NA, in a fashion similar to other polymeric nanocarriers, facilitate the passive targeting of AmB via macrophages to the site of infection; this may explain the enhanced antifungal efficacy (Fig. 6).

## Conclusion

In the changing scenario of fungal pathogenesis, improving the efficacy of the ‘gold standard’ antifungal drug AmB, is an area of active research. We report a green synthesis protocol for AmB super-aggregation employing *Aloe vera* leaf extract. The strategy provides an altogether novel approach to improve the therapeutic index of the antibiotic. The as-synthesized AmB nano-assemblage was stable; released the drug slowly in requisite dosage in sustained manner over an extended period of time and thereby minimizing interaction with serum components. Moreover, the biomimetic AmB-NAs were strategically more efficient as compared to the existing commercial formulation, Fungizone; whereas their toxicity to mammalian cells as well as experimental animals was significantly reduced. While excipients are generally used for fabrication of nano-sized drug formulation, the present approach offers an excipient free nano-formulation of AmB. Moreover, the transformation of parent drug molecules to nano-size dimension can result in their high uptake into the reticuloendothelial system. We envisage that biomimetically fabricated AmB nano-assemblage has taken us one step closer towards solving the growing demand of an active, less toxic substitute of AmB based formulations.

## Methods

### Preparation of Aloe vera extract

Thoroughly washed *Aloe vera* leaves (30 g) were finely cut and boiled in 100 ml sterile distilled water as described earlier^30,36^. The boiled extract was cooled and filtered through Whattman filter and the filtrate was stored at −20 °C till further use.

### Synthesis of AmB-nanoassemblies

Increasing volumes (1–5 mL) of *Aloe vera* leaf extract (30% w/v stock solution in deionized water) was added to 5 mL of 1 mM AmB solution and the volume made up to 10 mL by deionized water. The mixture was incubated for specified time period at room temperature and centrifuged at 20,000 g at 4 °C to pellet the AmB-NAs. Aliquots from the cocktail were withdrawn at various time intervals for spectro-photometric analysis. Changes in the AmB super-aggregate formation was monitored in the presence of increasing concentrations of *Aloe vera* extract (1 mL, 3 mL and 5 mL). In another set of experiments, effect of AmB concentration on the formation of super-aggregate was evaluated by varying the AmB concentration from 0.5 mM to 2 mM, while *Aloe vera* leaf extract (5 mL) was kept constant. The effect of repeated washing steps on spectral properties of the super-aggregates was also assessed by comparing absorbance spectra before and after washing. The AmB-NAs, thus formed, were pelleted at 20,000 g at 4 °C and suspended in 1 mL of deionized water. Finally, the solution was freeze-dried without a cryoprotectant to yield a yellow fluffy product. They were further characterized by employing various spectrophotometric methods.

### Characterization of AmB-nanoassemblies

UV-Visible and fluorescence spectroscopic analysis were employed to ascertain the aggregation state of nano-assemblies. Zeta Potential (ζ-potential) of the nano-particles was determined using DTS software (Malvern Instrument Limited, UK) based on M3-PALS technology. TEM analysis was performed to analyze size and shape of the nano-aggregates. The hydrodynamic particle size of the AmB-NA were determined by DynaPro-TC-04 dynamic light scattering equipment (Protein Solutions, CA) equipped with a temperature-controlled microsampler. The structural characterization of both free as well as nano-particulate form of the drug was performed by X-ray diffraction (XRD). FTIR spectroscopic studies of AmB nano-particles were carried out employing a Perkin-Elmer FTIR Spectrum One spectrophotometer. GC-MS analysis of Aloe vera extract was performed by use of SHIMAZDU QP2010, Column Rtx-5 MS.

### *In vitro* release kinetic studies

The *vitro* release of AmB from AmB-NAs in surrounding milieu (PBS, Histidine buffer and serum) was evaluated following published protocol^69^ as standardized in our lab.

### Drug toxicity studies

- Toxicity of AmB formulations against human red blood cells (RBC)

### Antibiotic-induced RBC lysis

To study extent of haemolysis, a known number of RBCs (approximately 2 × 10^8^ cells/mL) were incubated with 1 mL of various AmB nano-formulations (containing 1, 5, 10, 50, 100 and 200 μg/mL AmB equivalents in final volume of 2 mL at 37 °C for 24 hour. Free form of AmB was dissolved in 50 μL of DMSO and the volume was made up to 1 mL with PBS (final 5% DMSO). After stipulated time, the reaction mixture was centrifuged at 1200 g and supernatant was collected its absorbance recorded at 576 nm for released haemoglobin. Triton X-100 (nonionic surfactant) at a concentration of 0.1% was used as a positive control for 100% cell lysis. Each data point represents mean ± SD (triplicate determination).

### Efflux of K^+^ from RBC

Intra-cellular K^+^ loss was measured after incubation of RBC (2 × 10^8^ cells/mL) with free AmB (pure drug) as well as AmBisome, Fungizone or various AmB-NAs (AmB-NA3, AmB-NA5) at a concentration of 0.5–100 μg/mL in PBS (total volume was 2.0 mL). After 4 h incubation, an aliquot of 200 µL was collected and centrifuged at 2,800 g for 2 min. The pellet was washed twice with saline solution and the cells were then lysed by addition of 15 mM LiNO_3_. The lysate was centrifuge at 11,200 g for 10 min and concentration of K^+^ was determined in the supernatant by flame photometry (Corning Flame Photometer Model 400, Acton, MA, USA). All assays were carried out in triplicates and the results are expressed as mean ± SD.

### Lactate dehydrogenase (LDH) release assay

J774A.1 cells were grown to a density of 10^5^ cells/mL in a culture medium consisting of DMEM plus 2 mM glutamine supplemented with 10% FCS at 37 °C, 5% CO_2_ and subsequently exposed to test samples at concentrations up to 100 μg/mL. After 24 h incubation, cell-free culture supernatant was collected and transferred to another plate. To each well, substrate mixture (100 μL) from the LDH assay kit (Roche) was added and then incubated for a further 30 min at 37 °C. The LDH release was detected by measuring the absorbance at 500 nm. The results are the mean of three independent experiments.

b) *In-vitro* cytotoxicity test
  The cytotoxicity of AmB-NAs was assessed against two mammalian cell lines, viz. HEK-293 and J774A.1 by determining the number of viable cells employing MTT (3-(4,5-dimethylthiazol-2-yl)-5-(3-carbo xy methoxy phenyl)-2-(4-sulfophenyl)-2H-te trazolium) method^86^.
c) Liver and kidney function tests

*In-vivo* studies were conducted following the protocol formulated by the Institutional Animals Ethics Committee of Aligarh Muslim University, Aligarh. Proper and humane care of animals was taken during study period. Female Balb/c mice (weighing 20–30 gm) were divided into five groups consisting of 10 animals each.

1. Group I, Control (only PBS)
2. Group II, AmBisome
3. Group III, Fungizone
4. Group IV, AmB-NA3
5. Group V, AmB-NA5

A single injection of AmB-nano drug (containing 1, 5, 10 and 15 mg/kg b.w. AmB equivalent in 200 µl), was administered via the lateral tail vein to healthy mice. Twenty-four hours later, blood was collected from the experimental mice to isolate plasma. Serum chemistry analysis for investigation of creatinine, Alanine aminotransferase (ALT), Aspartate aminotransferase (AST) and blood urea nitrogen (BUN) was performed by detection as per the manufacturer’s guidelines. The data recorded are the mean of three experiments carried out independently.

d) Hematological parameters

Female Balb/c mice (weighing 20–30 gm) were divided into five different groups each consisting of 6 animals. Treatment was commenced from day 1. All groups except PBS (control, group 1) received a daily dose of 1 mg/kg bw of AmB equivalents up to day 7 via the lateral tail vein. The Fungizone group (group 2) received daily dose of 1 mg/kg bw of AmB as a solution in DMSO (5% w/v). Group 3 & 4 were administered with AmB-NA3 and AmB-NA5 formulations respectively whereas the AmBisome group (group 5) received marketed liposomal preparation of AmB (Gilead Sciences Inc., CA, USA). On day 8 (i.e. one day after final dose), mice from each group were subjected to ketamine anaesthesia and bled from retro-orbital plexus. Then, 10 µL of each blood sample was processed in a POC blood analyser ABX-MicrosCRP200 (Horiba Medical, Montpellier, France), to determination white blood cell (WBC) count, red blood cell (RBC) count, platelet count (PLT), haemoglobin (Hb) concentration and haematocrit (Hct)^87^. The results recorded are the mean value of three independent experiments.

### *In vitro* stability studies

AmB-NA5 was used as a representative AmB formulation and stored as freeze dried powder for 30 days at 37 °C/4 °C or as a sterile solution (1 mg/ml AmB equivalent) protected from light for 7 days at 37 °C/4 °C. At a predetermined time, an aliquot of the solution was passed through a 0.22 μm filter and AmB-NA5 concentration determined by HPLC. Dynamic Light Scattering was used to evaluate the change in size distribution over the period of study. RBC lysis test was used to determine the effect of storage on toxicity of AmB-NAs.

### Determination of maximum tolerated dose in healthy mice

The maximum tolerated dose (MTD), of various AmB-NA formulations was determined following the published protocol. Briefly, Balb/c mice (10 per group) were given a single dose (0.2 ml) of the AmB-NA via the tail vein (1 to 15 mg/kg bw AmB equivalent) diluted in PBS. Mice were thereafter observed for mortality, if any for 7 days. Mortality occurring within 1 h after drug administration was considered immediate death. Mice that survived for 96 h were sacrificed on day 30. Blood urea nitrogen and serum creatinine, as parameters for renal toxicity, and aspartate aminotransferase and alanine aminotransferase, as parameters for liver toxicity, were determined in serum samples of mice sacrificed 24 h or 7 days after termination of treatment. The maximum tolerated dose (MTD) was defined as the maximum dose that did not result in death or a > 3-fold increase in the indices for renal and liver function compared to indices of untreated mice^20^.

### *In vitro* susceptibility testing

The broth microdilution method, as recommended by the CLSI, was employed to determine anti-*Candida* activity of various AmB formulations^88^. The broth dilution tests were performed at 35 °C, and MICs were determined after 48 h incubation by observation of the presence or absence of visible growth. For AmB, MIC endpoint is the lowest concentration that inhibits visual growth or an endpoint score of 0 (100% inhibition). MFCs was determined by seeding the total volume from well with MIC against the highest inoculum onto Sabouraud dextrose agar (SDA) plates. All assays were performed in triplicate and repeated at least thrice.

### Fungicidal assay

*C*. *albicans* was grown overnight at 35 °C on Sabouraud dextrose agar (SDA). The colonies were suspended in 0.9% NaCl to get a turbidity equivalent of 0.5 McFarland Standard. Wells containing RPMI 1640 (buffered with 0.165 M MOPS to pH 7.0) plus AmB-NA or Fungizone at (4 × ) MIC or no antibiotic (growth control) were seeded with the yeast suspension to a final concentration of ~10^5^ cfu/ml. The cultures were incubated at 35 °C for up to 24 h. At the stipulated time periods, aliquots were removed and the number of viable cells per milliliter was determined by counting colonies (colony forming units per ml, CFU/ml) on SDA after serial dilutions in PBS. The assay was performed in quadruplicate. Each result is representative of at least three separate experiments.

### Preparation of inoculum and induction of experimental candidiasis

For *in vivo* infection purpose, *C*. *albicans* strain (ATCC 18804) was cultured in 5% dextrose broth at 37 °C for 24 hours. The cell suspension was centrifuged at 5000 g for 15 min at 4 °C followed by washing with sterile normal saline. The number of cells was counted using haemocytometer.

Induction of experimental candidiasis was done following published procedure as standardized in our lab^89^. Briefly, each mouse was challenged via the tail vein with 1 × 10^7^ Colony Forming units (CFU) of *C*. *albicans* (ATCC 18804) suspended in 200 µl of sterile normal saline (150 mM, pH 7.4). This inoculum size was found to consistently produce a disseminated infection within 48 h after injection and ultimately ensuing in death of animals within 7 days post challenge with infection in pilot studies (data not shown).

### Assessment of antifungal efficacy of AmB nano-assemblage

The efficacy of AmB-NA was assessed by monitoring the survival of the infected animals and determining the clearance of *C*. *albicans* from various vital organs viz. liver, spleen and kidney. For survival and residual fungal burden studies, two separate but similar experiments were set up simultaneously. In each experiment, the infected animals were divided in 5 different groups as listed below. Each group had 20 animals.

1. Group I, Control (Only PBS)
2. Group II, Free AmB
3. Group III, Lip AmB
4. Group IV, AmB-NA3 (prepared with 3 ml of extract)
5. Group V, AmB-NA5 (prepared with 5 ml of extract)

The animals in Group I (only PBS) were not given any drug treatment. Free form of AmB (dissolved in 5% DMSO) was administered at dose of 5 mg/kg b.w. in animals of Group II.

For survival studies, mortality of the animals was observed twice each day, during 50 days of observation period. Quantitative assessment of the fungal burden in various vital organs such as the liver, spleen and kidney was performed as described elsewhere^86^. Briefly, antifungal treatment was begun 24 hours after challenging the animals with *C*. *albicans* infection. The animals (three from each group) were sacrificed on day 7, 15 and 21 post-infection. The vital organs viz. liver, spleen and kidney were taken out aseptically. The organs were washed extensively with hypotonic buffer, homogenized and serially diluted with normal saline. Various dilutions of each homogenized organ were dispersed on YPD agar plates containing gentamycin to avoid bacterial contamination. After incubation of 12–24 hours at 37 °C, the colonies were counted and the fungal load was calculated by multiplying with the dilution factor.

### Ethics Statement

All animal experiments were approved by the Institutional Animal Ethics Committee of IBU-AMU, India. Experiments involving bleeding, injection and sacrifice of animals were strictly performed according to the National Regulatory Guidelines issued by the Committee for the Purpose of Control and Supervision of Experiments on Animals (CPCSEA), Government of India (http://moef.nic.in/division/committee-purpose-control-and-supervision-experiments-animals-cpcsea-1). Our approval ID was 332/CPCSEA.

### Statistical analysis

Data from various experimental groups were compared employing student’s t-test, and one way ANOVA (Holm-Sidak method) to assess level of significance in observed data from various groups, using Sigma-Plot version 10 software. P values < 0.05 were considered to be significant.

## Electronic supplementary material

Supplementary Information
