# Supplementary material for: Biomimetically engineered Amphotericin B nano-aggregates circumvent toxicity constraints and treat systemic fungal infection in experimental animals
Source: Sci Rep. 2017 Sep 19;7:11873. doi: 10.1038/s41598-017-11847-0 (PMC5605718; doi:10.1038/s41598-017-11847-0)
Supplement: Supplementary file 1 — Supplementary Information [file 41598_2017_11847_MOESM1_ESM.pdf]

Biomimetically engineered Amphotericin B nano-aggregates circumvent toxicity constraints and treat systemic fungal infection in experimental animals

Qamar Zia[1], Owais Mohammad[1], Mohd Ahmar Rauf[1], Wasi Khan[2], Swaleha Zubair[3,*]

[1]Interdisciplinary Biotechnology Unit, Aligarh Muslim University, Aligarh, INDIA

[2]Department of Applied Physics, Aligarh Muslim University, Aligarh, INDIA

Women's College, Aligarh Muslim University, Aligarh, INDIA

***Name and address of corresponding author:**

Swaleha Zubair, Women's College, Aligarh Muslim University, Aligarh, U.P.-202002, INDIA.

E-mail: swalehazubair@yahoo.com

Supplementary Materials and Methods

Experimental Procedures

Chemicals and reagents

Amphotericin B (European Pharmacopoeia (EP) Reference Standard), RPMI 1640, DMEM, dimethyl sulfoxide (DMSO) and dialysis membrane (MWCO 3 kD) were obtained from Sigma Aldrich, USA. The antifungal agents, AmBisome (Gilead Sciences Inc., San Dimas, CA, USA) and Fungizone (Apothecon, Princeton, NJ, USA) were re-constituted according to the manufacturer's instructions. Sterile filters of 0.22µm size were purchased from Millipore, India. Tissue culture plastic-wares were bought from BD Biosciences (USA). Kits for liver and kidney function tests were from Span Diagnostics, India. HEK-293 cells (human embryonic kidney cells) and mouse J774A.1 cells (monocyte macrophage cell line derived from a tumor in a female BALB/c mouse) were procured from ATCC, Manassas, VA, USA. All other chemicals were of analytical grade and acquired locally.

Fungal isolates

Various test strains of *Candida albicans* (ATCC 18804, ATCC 90028), *C. glabrata* 4224A, *Apergillus fumigatus* (ATCC 9157) were obtained from ATCC, USA while *C. glabrata* (MTCC 3019) was procured from MTCC, Chandigarh, India. Sabouraud dextrose agar/broth (SDA) was used for growing all the fungal strains. Both *C. albicans* and *C. glabrata* strains were characterized using Hi-CHROME agar as well as germ tube formation using standard protocol prior to their use in various studies. *C. albicans* (ATCC 10231) was used as quality control strain.

Animals

Inbred female Balb/c mice of 20±2 g weight, were obtained from the animal facility at Indian Institute of Toxicological Research (IITR), Lucknow, India. The animals were acclimatized for a few days under standard husbandry conditions: room temperature (22±3°C), relative humidity (65±10%) and 12 hour light/dark cycle. They had free access to standard dry pellet diet and water under strict hygienic conditions. Humane endpoints were applied for mice which survived at the end of the experiments. Animals were intraperitoneally administered with anesthetic cocktail of ketamine (5mg/kg) and xylazine (4 mg/kg) prior to sacrifice and then euthanized via cervical dislocation. In all experimental procedures, efforts were made to minimize pain and suffering of the animals. All animal experiments were reviewed and approved by the Institutional Animal Ethics Committee, AMU, Aligarh, India.

Determination of particle size and ζ-potential

The cumulative mean of AmB-NAs size, polydispersity index (PDI) and zeta potential of AmB-nanoassemblies were determined by dynamic light scattering using Zeta sizer (Nano ZS, Malvern Instruments, Worcestershire, U.K.) after suitable dilution (1:1000). The zeta potential (ζ-potential) gives an indication of the charge acquired by particulate system on its suspension in aqueous medium. Samples were filtered using 0.22 µm syringe filter (PES, Millipore) to remove contamination with dust particles. The formulation was lyophilized in a 2.0 ml microfuge tube and the samples were reconstituted in 20 mM phosphate buffer (pH 7.4). The zeta sizer measures the electrophoretic mobility of the particles, which was converted into the zeta potential using the Helmholtz−Smoluchowski equation built into the

Malvern Zeta-sizer software. The experiments were repeated five times and the average ζ-potential and standard deviations were calculated. For size measurements, samples were diluted in MilliQ water and measured for at least 120s. The concentration of AmB−NAs used was 10^{-4} M. All of the measurements were performed 5 times and average values were taken.

X-ray diffraction analysis

The structural characterization of both free as well as nanoparticulate form of the drug was performed by X-ray diffraction (XRD). All the diffraction patterns were prepared as step-scans. XRD was performed in the 2θ range of 20-80° (RigakuMiniflex II) with Cu-K_α radiations (λ = 1.5406 Å) operating at voltage of 30 kV and current of 15 mA. The scan was smoothed with a weighted moving average to obtain a diffractogram as displayed in the results section. The vertical axis records X-ray intensity and the horizontal axis records angles in degrees 2-theta.

Fourier transform infrared spectroscopy (FT-IR) measurement

FTIR spectroscopic studies of AmB nanoparticles were carried out employing a Perkin-Elmer FTIR Spectrum One spectrophotometer in the diffuse reflectance mode operating at a resolution of 4 cm^{-1}. Each sample was scanned three times to check the authenticity of data. The spectra were taken between 4000 cm^{-1} and 1000 cm^{-1} by averaging 128 scans for each spectrum.

Dynamic Light Scattering (DLS) Measurements

DLS measurements were carried out using DynaPro-TC-04 dynamic light scattering equipment (Protein Solutions, Wyatt Technology, Santa Barbara, CA) equipped with a temperature-controlled microsampler. The AmB-NAs (2 mg/ml) were filtered serially through 0.22 and 0.02 µm Whatmann syringe filters directly into a 12 µl quartz cuvette. For each experiment, 20 measurements were taken. Mean hydrodynamic radius (R_h) was analyzed using Dynamics software at optimized resolution. The R_h was estimated on the basis of an autocorrelation analysis of scattered light intensity data based on translation diffusion coefficient by Stoke's-Einstein relationship-

$$R_h = \frac{k\,T}{6\pi\eta D}$$

where, R_h is the hydrodynamic radius, k is Boltzmann constant, T is temperature, η is the viscosity of water and D is diffusion coefficient.

Transmission electron microscopy

TEM was performed to characterize the size and surface morphology of AmB NAs using the electron microscope (model Philips CM-10, Germany). For TEM studies, AmB−NAs was used at a concentration of 10 µg/mL AmB equivalents in double distilled water. The lyophilized preparation of nanoparticles was suspended in 20 mM PBS (pH 7.4) and a drop of the formulation was mounted on clear glass stub, air dried and coated with gold-palladium alloy using a sputter coater. An accelerating voltage of 20 kV was used for imaging.

In vitro release kinetic studies

The *vitro* release of AmB from AmB-NAs in surrounding milieu (PBS and serum) was evaluated following published protocol[1] as standardized in our lab. Briefly, a stock solution of AmB-NAs was prepared in water at a concentration of 1mg/ml AmB equivalent. Then 1 ml of this aqueous solution was added to either 1ml of sterile PBS, pH 7.4 (20 mM, with 5%

DMSO), serum (1 ml, containing 5% DMSO). The final concentration of AmB-NA was 500µg/ml AmB equivalents (total amount of AmB was 1mg in 2ml). The incubation studies were performed in the presence of 0.02% (w/v) of sodium azide to avoid the microbial growth. The AmB-NA + buffer solution (2ml) was placed in a dialysis bag having MWCO 3-4 kDa and dialyzed against 50 ml of PBS/DMSO (pH 7.4, 95:5%, v/v) at a speed of 50 rpm at 37 ± 0.5°C. Samples (500 µl) were collected at regular intervals for drug concentration analysis and medium was replaced by fresh buffer (PBS/DMSO; 95:5%, v/v) to prevent drug saturation (maintaining strict sink conditions throughout the experiment). Next, the release kinetic study was also determined in histidine buffer (pH 6.0). Release runs were continued for 240 h. The AmB content was determined by measuring absorbance in aqueous methanol (50% v/v) at 405 nm by HPLC. The amount of AmB released was then calculated from the calibration curve of AmB plotted in aqueous methanol (50% v/v). The results recorded are the mean value of three independent runs.

HPLC Analysis

Quantitation of AmB was performed by a slight modification of method described by Nilsson-Ehle[2] as standardized in our lab. High Performance Liquid Chromatography was conducted using Micro Bondapak C-18 column (30cm X 4mm [diameter]; particle size 5 µm; Waters Associates) using Waters HPLC system. The mobile phase was a mixture of methanol and 0.005 M EDTA (8:2 vol/vol) deaerated by vaccum. The injection volume was 20 µl delivered at a rate of 1.0 ml/min. The eluent was monitored at 405nm for AmB content with a run time of 15min. The retention time of AmB was 8 min. The internal standard was 1-amino-4-nitronaphthalene. The limit of detection was 1 ng. The results were quantified by comparison of AmB peak at 405nm with a standard of known concentration eluted at the same retention time. A standard calibration curve of the drug was plotted at 405 nm by determining the area under curve corresponding to the increasing amount of the drug (3.125ng-200ng of AmB equivalent in 20µl sample injection volume). The highest AmB concentration was 10µg/mL (equivalent to 200ng of AmB in 20µl). Dilutions of AmB were performed in methanol to ensure that AmB remained in the monomeric form.

GC-MS analysis

GC-MS analysis of Aloe vera extract was performed by use of SHIMAZDU QP2010, Column Rtx-5 MS[3] (30 meter X 0.25 mm i.d.X 0.25 um film thickness), oven temperature 50˚C to 280˚C at 4˚C/min, for 5 min; inlet and interface temperatures were 250˚C and 280˚C, respectively. Carrier gas was He at a flow rate of 1.0 ml/min (constant flow). 0.2 ml of sample was injected under split of 20:1. EIMS: electron energy, 70 eV. Interpretation of mass spectrum GC-MS was conducted using data base of NIST, having more than 62,000 patterns. The spectrum of the known compounds was compared with the NIST library.

Drug toxicity studies

a) **Toxicity of AmB formulations against human red blood cells (RBC)**

Isolation of human erythrocytes:

Fresh blood (15–20 ml) was kindly provided by the Blood Bank, Jawaharlal Nehru Medical College, Aligarh Muslim University, Aligarh and collected in anticoagulant solution (EDTA 1 mg/ml, at 10% w/v). The blood was centrifuged at 1000g for 15 minutes at 4°C. Plasma was carefully aspirated and the exposed buffy coat was removed and discarded. The RBCs were washed three times by centrifugation (1,000 g for 15 minutes at 4°C) and suspended in

5 volumes of normal saline (0.9% NaCl). The packed cell were then resuspended in 4 ml of saline and counted using Neubauer™ chamber (Splabor, São Paulo, Brazil).

Antibiotic-induced RBC lysis

To study extent of haemolysis, a known number of RBCs (approximately 2×10^8 cells/ ml) were incubated with 1 ml of various AmB nano-formulations (containing 1, 5, 10, 50, 100 and 200 µg/ml AmB equivalents in final volume of 2 ml) at 37°C for 24 hour. Free form of AmB was dissolved in 50 µl of DMSO and the volume was made up to 1 ml with PBS (final 5% DMSO). After stipulated time, the reaction mixture was centrifuged at 1200g, supernatant was collected and its absorbance was recorded at 576nm for released haemoglobin. Triton X-100 (nonionic surfactant) at a concentration of 0.1% was used as a positive control for 100% cell lysis. Fungizone and AmBisome solutions prepared as mentioned in the manufacturer's protocol in double distilled sterile water was included as control .The result was expressed graphically as percentage of 100% cell lysis and was determined by the following equation:

$$\textbf{Percent Hemolysis} = \frac{Abs_T - Abs_C}{Abs_{100\%} - Abs_C} \times 100$$

where; Abs_T is the absorbance of the supernatant from samples incubated with various AmB formulations, Abs_C is the absorbance of the supernatant from control (PBS) and $Abs_{100\%}$ is the absorbance in the presence of 0.1% Triton X-100. Results are mean of three independent experiments. The toxicity effect of Aloe vera extract (at a concentration of ~0.5g/ml) on RBC lysis was also studied.

Efflux of K^+ from RBC

Intra-cellular K^+ loss was measured after incubation of RBC (2×10^8 cells / ml) with free AmB (pure drug) as well as AmBisome, Fungizone or various AmB-NAs (AmB-NA3, AmB-NA5) at a concentration of 0.5–100 µg/ml in PBS (total volume was 2.0 ml). After 4 h incubation, an aliquot of 200 µl was collected and centrifuged at 2,800g for 2 min. The pellet was washed twice with saline solution and the cells were then lysed by addition of 15 mM $LiNO_3$. The lysate was centrifuge at 11,200 g for 10 min and concentration of K^+ was determined in the supernatant by flame photometry (Corning Flame Photometer Model 400, Acton, MA, USA). The baseline level (0% release) was defined as the amount of K^+ released from the RBCs after incubation with PBS buffer (negative control); 100% release was defined as the amount of K^+ released from the RBCs after incubation with 10 µM valinomycin (Calbiochem, La Jolla, CA) (positive control). The remaining K^+ was calculated as a percentage of the total content released by positive control (10 µM valinomycin). For each agent (AmBisome, Fungizone and AmB-NA), the concentration of AmB that produced 50% release of K^+ from the RBCs (K_{50} value) was then calculated. Results are given as the mean of three experiments carried out with each concentration in triplicate.

Lactate dehydrogenase (LDH) release assay

J774A.1 cells were grown to a density of 10^5 cells/ml in a culture medium consisting of DMEM + 2mM glutamine supplemented with 10% FCS at 37°C, 5% CO_2 and subsequently exposed to test samples (PBS, AmB-NA3, AmB-NA5, Fungizone® and AmBisome®) at concentrations up to 100 µg/ml. After 24 h incubation, cell-free culture supernatant was collected and transferred to another plate. To each well, substrate mixture (100 µl) from the LDH assay kit (Roche) was added and then incubated for a further 30 min at 37°C and 5% CO_2. The LDH release was detected by measuring the absorbance at 500 nm. Triton X-100

(0.1%) was used as a positive control. The results recorded are the mean value of three runs carried out independently.

b) In-vitro cytotoxicity test

The cytotoxicity of AmB-NAs was assessed against two mammalian cell lines, viz. HEK-293 and J774A.1 by determining the number of viable cells employing MTT (3-(4,5-dimethylthiazol-2-yl)-5-(3-carboxy methoxy phenyl)-2-(4-sulfophenyl)-2H-tetrazolium) method[4]. HEK-293 and J774A.1 cells were cultured overnight in 96-well flat bottom tissue culture plates (Nunc, USA) at a density of 1×10^5 cells/ml in DMEM (with 2mM glutamine + 10% FCS) (Sigma-Aldrich). Next morning, the cells were treated with 100 µl of AmB-NAs at various concentrations (1-200 µg/ml AmB equivalents) in triplicate. Fungizone, AmBisome and pure AmB (free form) were used as controls. The plates were incubated for 24 h at 37°C, 5% CO_2. After washing the cells, 200 µl of 0.5 mg/ml of MTT solution in DMEM was added to each well and incubated at 37°C for 2h. The supernatant was removed and 200 µl extraction solution (DMSO) was added to solubilize the formazan crystals. After 30 min incubation at room temperature, the absorbance of each well was measured at 550 nm and the percentage of viable cells was calculated using the following formula:

$$\textbf{\% Viable cells} = \frac{\textbf{Sample absorbance - blank absorbance}}{\textbf{Control absorbance - blank absorbance}} \textbf{ X 100}$$

Where blank absorbance corresponds to media alone and control absorbance is cells with media alone. All assays were carried out in triplet and results are expressed as mean.

c) Liver and kidney function tests

In vivo studies were conducted following the protocol formulated by the Institutional Animals Ethics Committee of the University. Proper and humane care of animals was taken during study period. Female Balb/c mice (weighing 20-30 gm) were divided into five groups with 10 animals per group.

(1) Group I, Control (only PBS)

(2) Group II, AmBisome

(3) Group III, Fungizone

(4) Group IV, AmB-NA3

(5) Group V, AmB-NA5

A single injection of AmB-nano drug (containing 1, 5, 10 and 15 mg/kg b.w. AmB equivalent in 200 µl), was administered via the lateral tail vein to healthy mice. Fungizone and AmBisome were administered at a dose of 2 mg/kg b.w. and 10 mg/kg b.w. respectively. Twenty-four hours later, blood was collected by retro-orbital puncture from the mice belonging to various experimental groups into lithium heparin Microtainers® (BD, Franklin Lakes, NJ, USA) and centrifuged at 2,000 g, 4°C for 10 min to isolate plasma. No animal was bled more than once for this purpose. Samples were frozen at −80°C until use. Serum chemistry analysis for investigation of creatinine, Alanine aminotransferase (ALT), Aspartate aminotransferase (AST) and blood urea nitrogen (BUN) was performed by detection as per

the manufacturer guidelines. Data recorded are mean of three experiments carried out independently.

In vitro stability studies

AmB-NA5 was used as a representative AmB formulation and stored as freeze dried powder for 30 days at 37°C/4°C or as a sterile solution (1 mg/ml AmB equivalent) protected from light for 7 days at 37°C/4°C. At a predetermined time, an aliquot of the solution was passed through a 0.22 μm filter and AmB-NA5 concentration was determined by HPLC. Dynamic Light Scattering was used to evaluate the change in size distribution over the period of study. Lysis test was used to determine the effect of storage on the toxicity of AmB-NAs. The AmB-NA was also subjected, in duplicate, to three freeze thaw cycles of 24 h each by freezing at a temperature of −20°C and thawing at a temperature of 32°C following which NAs were examined for change in size.

***In vitro* susceptibility testing**

A stock solution (1mg/ml) of amphotericin B was prepared in DMSO. The solution was further diluted with DMSO and subsequently with the appropriate test medium. Amphotericin B was tested over a concentration range of 0.015 to 8 μg/ml. *C. albicans* ATCC 10231 was used as a quality control strain.

The broth microdilution method was used as the reference method, as described by the Clinical and Laboratory Standards Institute (CLSI; formerly NCCLS) M27-A2 (Vol. 22 No. 15)[5]. The inoculums was prepared from Sabouraud dextrose agar subcultures incubated at 35°C for 24h and the resulting suspension was adjusted spectro-photometrically to a density equivalent to a 0.5 MacFarland standard at 530 nm (1.5 X 10^6 CFU/ml). A working suspension was made by 1:1000 dilution of the suspension in RPMI medium with L-glutamine and without bicarbonate (Sigma Aldrich), buffered to pH 7.0 with 0.165 M morpholine propane sulfonic acid (MOPS; Sigma). After that, 100 µl of the diluted inoculum was added to each well. The final inoculum size was 0.5–2.5 X 10^3 CFU/ml. The broth dilution tests were incubated at 35°C, and MICs were determined after 48 h incubation by observation of the presence or absence of visible growth. For AmB, MIC endpoint is the lowest concentration that inhibits visual growth or an endpoint score of 0 (100% inhibition).

After determination of MICs, the entire contents (200 µl) of each well with drug concentrations above the MIC were plated onto two drug free SDA plates (100 µl aliquots/plate). Aliquots were placed in a single spot on the agar plate and, after drying, the cells were dispersed by streaking. Plates were incubated at 35°C for 48 h; the MFC was the lowest drug concentration that showed either no growth or fewer than three colonies to obtain approximately 99 to 99.5% killing activity. All assays were performed in duplicate and repeated at least twice.

References

1. Zia, Q., Khan, A. A., Swaleha, Z. & Owais, M. Self-assembled amphotericin B-loaded polyglutamic acid nanoparticles: preparation, characterization and in vitro potential against Candida albicans. Int. J. Nanomedicine 10, 1769 (2015).

2. Nilsson-Ehle I, Yoshikawa TT, Edwards JE, Schotz MC, Guze LB. Quantitation of amphotericin B with use of high-pressure liquid chromatography. The Journal of Infectious Diseases.135 (3):414-22 (1977).

3. Ali, K. et al. Aloe vera extract functionalized zinc oxide nanoparticles as nanoantibiotics against multi-drug resistant clinical bacterial isolates. J. Colloid Interface Sci. 472, 145−156 (2016).

4. Mosmann, T. Rapid colorimetric assay for cellular growth and survival: Application to proliferation and cytotoxicity assays. J. Immunol. Methods 65, 55–63 (1983).

5.Clinical and Laboratory Standards Institute (CLSI). Reference method for broth dilution antifungal susceptibility testing of yeasts; approved standard-method-Second Edition (ISBN 1-56238-469-4). Wayne, PA: CLSI. 2002; Vol. 22 (No. 15) (2002).

SUPPORTING INFORMATION

Legends to Supplementary Figures

Figure S1: Absorption spectra of the as-synthesized nano-AmB formulation (AmB-NA3 and AmB-NA5) in **(A)** organic solvent and **(B)** aqueous medium.

Figure S2: Effect of *Aloe vera* leaf extract fraction on biomimetic synthesis of AmB-NA: **(A)** The *Aloe vera* leaf extract does not lose its potential to synthesize AmB-NA upon treatment with DNase, RNase or Proteinase as revealed by UV-Vis absorption spectrum of as-synthesized AmB-NA. **(B)** Spectra of AmB-NA synthesized with *Aloe vera* leaf extract without dialysis or with *Aloe vera* leaf extract content obtained after dialysis with 2 Kd cut off membrane. **(C)** Spectra of AmB-NA synthesized in the presence of Aloin alone. **(D)** TEM micrograph of AmB-NA synthesized with Aloin.

Figure S3: Hemolytic effect of Aloe vera extract: From a stock solution of Aloe vera (concentration of ~0.5g/ml), variable amounts of the extract (containing 2, 4, 8, 16, 32, 64, 128 and 256 μl in final volume of 2 ml) were taken and incubated with a known number of RBCs (approximately 2×10^8 cells/ ml) at 37°C for 24 hour. After stipulated time, the reaction mixture was centrifuged at 1200g and supernatant was collected its absorbance recorded at 576nm for released haemoglobin. Each data point represents an average from three different experiments±SD.

Figure S4: Hemolysis assays after storage of the AmB−NA complex: (A) Hemolysis (24 h incubation) after storage of AmB−NA complex at 4°C for 30 days in a solid form. Each data point represents an average from three different experiments±SD. **(B)** Hemolysis (24 h) of AmB−NA complex after storage at 37 °C for 30 days in a solid form. Each data point represents an average of data pooled form three different experiments ± standard deviation. **(C)** Hemolysis after storage of AmB−NA complex from the same batches as used in panel (24 h incubation) at 37 °C for 7 days as a solution (1.0 mg/mL). Fungizone and AmBisome were used as control. Each data point represents mean±SD (quadruplicate determination).

Figure S5: GC-MS of Aloe vera extract.

SUPPORTING FIGURES

FIGURE S1

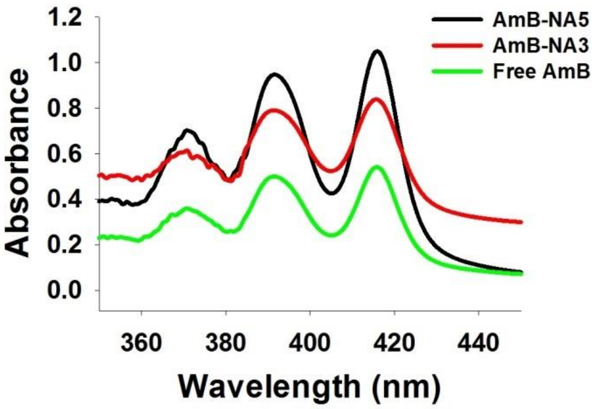

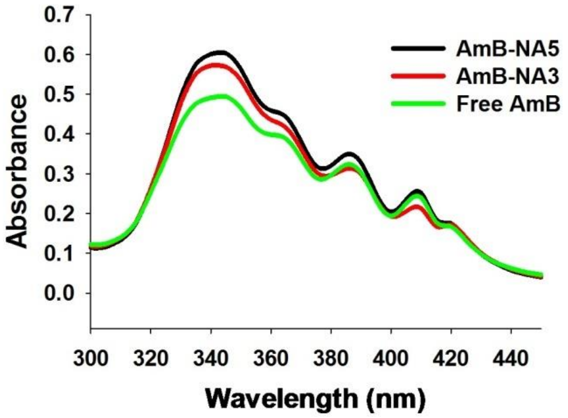

FIGURE S2

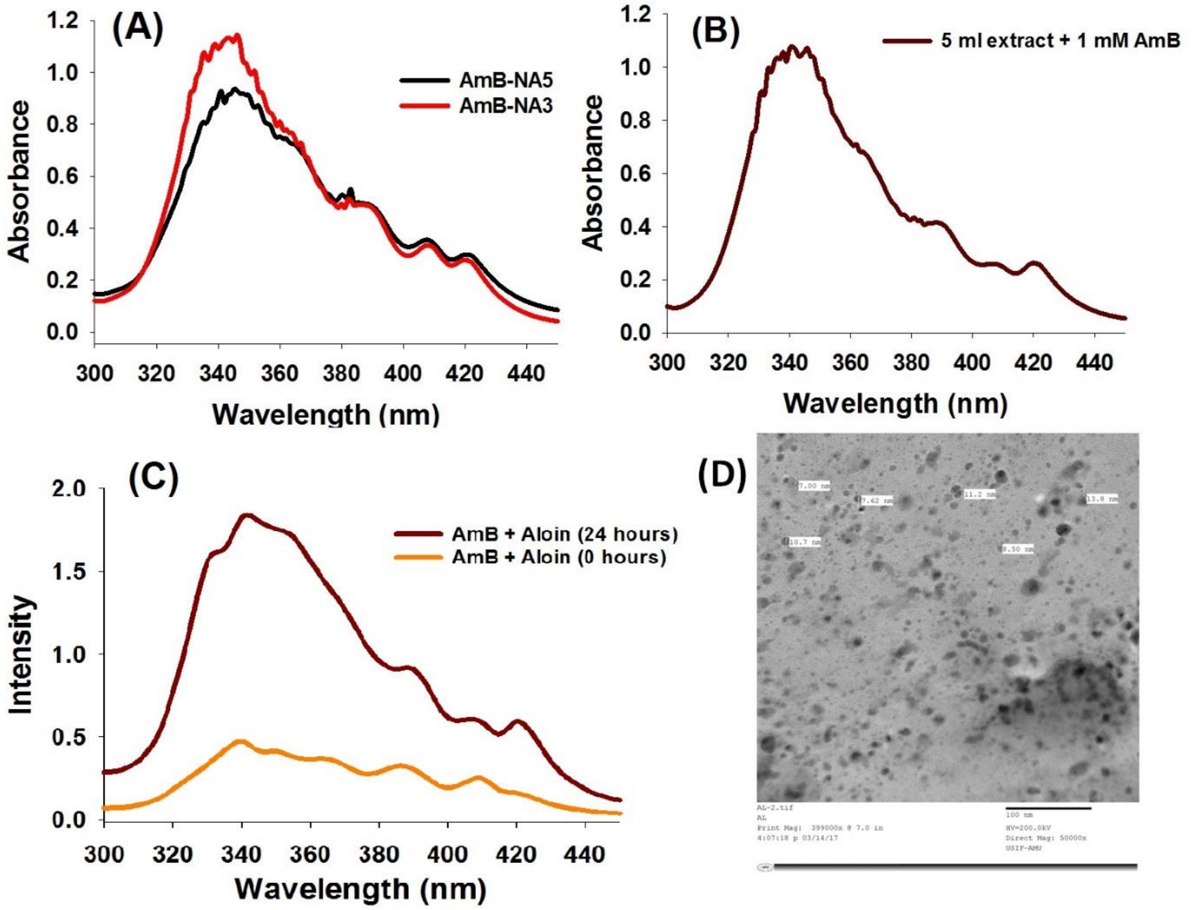
(A)
Absorbance
Wavelength (nm)
AmB-NA5
AmB-NA3
(B)
5 ml extract + 1 mM AmB
Absorbance
Wavelength (nm)
(C)
AmB + Aloin (24 hours)
AmB + Aloin (0 hours)
Intensity
Wavelength (nm)
(D)
7.00 nm
7.62 nm
11.2 nm
13.8 nm
10.7 nm
8.50 nm
AL-2.tif
AL
Print Mag: 399000x @ 7.0 in
4:07:18 p 03/14/17
100 nm
HV=200.0kV
Direct Mag: 50000x
USIF-AMU

FIGURE S3

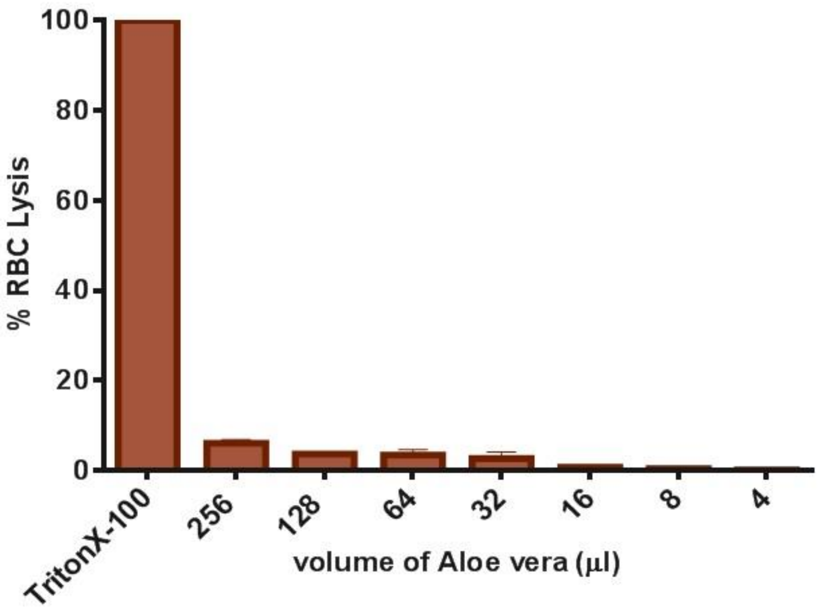

100
80
60
40
20
0
% RBC Lysis
TritonX-100
256
128
64
32
16
8
4
volume of Aloe vera (μl)

FIGURE S4

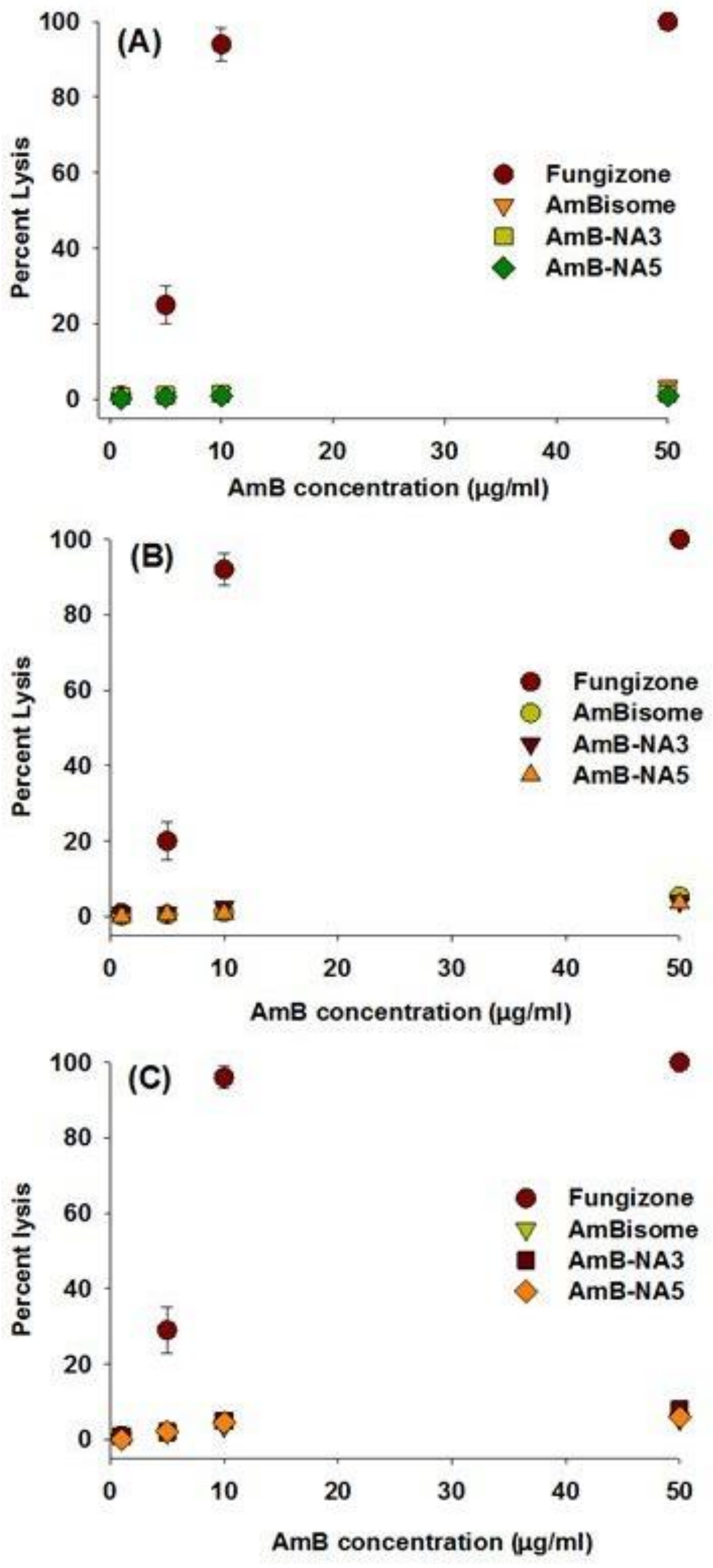
(A)
Percent Lysis
100
80
60
40
20
0
0
10
20
30
40
50
AmB concentration (µg/ml)
Fungizone
AmBisome
AmB-NA3
AmB-NA5
(B)
Percent Lysis
100
80
60
40
20
0
0
10
20
30
40
50
AmB concentration (µg/ml)
Fungizone
AmBisome
AmB-NA3
AmB-NA5
(C)
Percent lysis
100
80
60
40
20
0
0
10
20
30
40
50
AmB concentration (µg/ml)
Fungizone
AmBisome
AmB-NA3
AmB-NA5

FIGURE S5

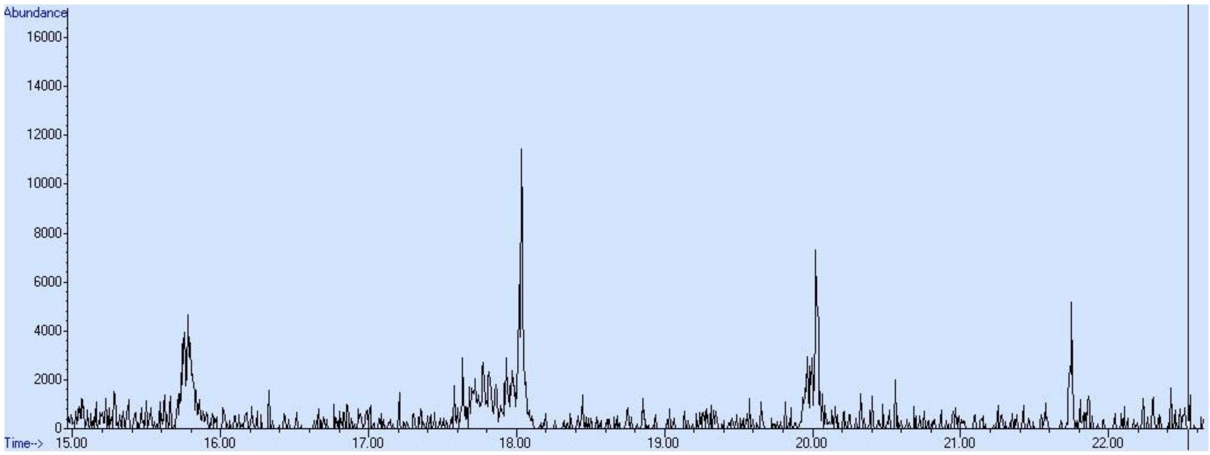
Abundance
16000
14000
12000
10000
8000
6000
4000
2000
0
Time-->
15.00
16.00
17.00
18.00
19.00
20.00
21.00
22.00

SUPPORTING TABLES

Table S1: Position of FTIR vibrations of AmB

Wavenumber [cm^{-1}]	Assignment*
994	glycosidic linkage + δ(-C-H)
1095sh	v_{as}(C–O)
1464	v_s($-COO^-$), polyene δ(-CH)
1579	δ_s($-NH_3^+$) + v_{as}($-COO^-$)<br>v(C=C)
1604, 1657	δ_{as}($-NH_3^+$), v(C=O) (aromatic ketone)
(2936) sh	v_{s+as}(CH_2, CH_3) + v(–CH)
3440, 3462	v(-OH), v(N-H) (Strongly H-bonded)

* v, stretching mode; δ, bending in plane; γ, bending out of plane; s, symmetric vibrations; as, asymmetric vibrations; sh, shoulder; () = weak, frequency ambiguous

Table S2: Stability study of sterile solution (1 mg/ml AmB) of AmB-nanoassembly (AmB-NA5)

Characteristics	0 days	7 days
**% AmB content**	100	95
**Size distribution (nm)**	107±10	110±9

Table S3: Effect of freeze thawing on the size of the formulation

Test Condition	Size (nm)	
	**AmB-NA3**	**AmB-NA5**
**Before freeze-thaw**	100±4	110±7
**After freeze thaw**	105±10	120±9

Table S4: Kinetic model of release profile of AmB-nano-formulations: The AmB release profile was analyzed using various kinetic models to know the release mechanism. The table shows Correlation co-efficient for various kinetic models.

Kinetic Model	Correlation co-efficient	
	AmB-NA3	AmB-NA5
Zero Order	0.7655	0.7997
First order	0.6847	0.793
Higuchi	0.9157	0.9334
Korsmeyer−Peppas (KP)	0.7159	0.7244
Hixon-Crowell	0.964	0.966
